# Association Between Perioperative Sleep Disorders and Post‐Operative Delirium in Cardiac Surgeries: A Systematic Review and Meta‐Analysis

**DOI:** 10.1111/jsr.70241

**Published:** 2025-11-08

**Authors:** Hesam Varpaei, Pallav Deka, Lorraine B. Robbins, Kousha Farhadi, Mathew Reeves, Fabrice Mowbray, Stuart F. Quan

**Affiliations:** ^1^ College of Nursing Michigan State University East Lansing Michigan USA; ^2^ College of Nursing Wayne State University Detroit Michigan USA; ^3^ Anesthesia, Critical Care, and Pain Management Research Center Tehran University of Medical Sciences Tehran Iran; ^4^ Department of Epidemiology and Biostatistics, College of Human Medicine Michigan State University East Lansing Michigan USA; ^5^ Division of Sleep and Circadian Disorders, Brigham and Women's Hospital Harvard Medical School Boston Massachusetts USA

**Keywords:** cardiac surgical procedures, cardiovascular diseases, delirium, post‐operative period, sleep

## Abstract

Post‐operative delirium (POD) is an acute deterioration in cognitive function and highly prevalent after cardiac surgery (CS; up to 55%). Perioperative sleep disorders (PSD) are also commonly noted in surgical patients (up to 60%). The primary aim of our systematic review is to determine the association between PSD and POD in CS patients during their hospital stay. We searched five databases (PubMed, CINAHL, Web of Science, Scopus, and EMBASE) to identify studies evaluating the association between PSD and POD amongst CS (any open‐heart CS) patients, without time and geographic restriction. Original articles that focused on adults undergoing cardiac surgeries and assessed sleep and POD were included. We conducted a meta‐analysis using a random effects model to determine the effect of sleep quality on POD. Thirty‐three studies were included (63% observational designs); most studies originated from China (33%). The most frequently used subjective and objective sleep assessment tools were the Pittsburgh Sleep Quality Index (PSQI) (33%) and polysomnography (18%). After pooling observational data, we identified an incidence of POD ranging from 3.6% to 73%. Increased PSQI scores (standard threshold > 5) were associated with a greater likelihood of POD occurrence (standardised mean difference [SMD] = 0.73, *p* > 0.05). Lower total sleep time (SMD = −0.68, *p* < 0.05) was associated with an increased risk of POD. Poor sleep quality, insomnia, and sleep‐disordered breathing are prevalent forms of PSD and are major risk factors for POD following CS. Additional research is warranted to clarify when sleep quality normalises after cardiac surgery and how targeted interventions can accelerate this recovery.

## Graphical abstract

Overview of studies included in the systematic review investigating the association between perioperative sleep disorders (PSD) and postoperative delirium (POD) in patients undergoing cardiac surgery.

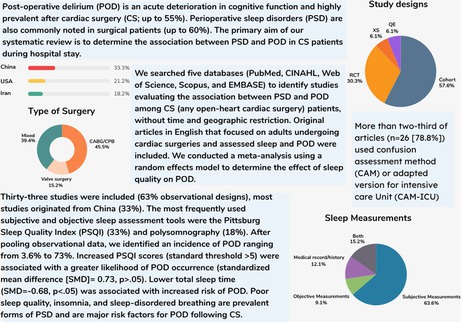

## 1 Introduction

Post‐operative delirium (POD) is a specific type of delirium that typically occurs within one to three days after surgery (Whitlock et al. 2011; Evered et al. 2018). The prevalence of POD has been found to be considerably higher in patients undergoing cardiac surgery with 55% experiencing this condition (Chen et al. 2021), compared to 24% in those undergoing noncardiac surgery (Igwe et al. 2023; Abate et al. 2021). A systematic review also noted a higher prevalence of POD in cardiac surgeries (32%), as compared to other kinds of surgeries (e.g., orthopaedic surgeries 20%, vascular surgeries 14%, spinal surgeries 13%, colorectal surgeries 14%) (Igwe et al. 2023).

Identifying POD is essential, as it is known to increase the risk of in‐hospital mortality (Lin et al. 2022), hospital re‐admission (de la Varga‐Martínez et al. 2023), and cognitive decline (Goldberg et al. 2020), whilst decreasing quality of life (de la Varga‐Martínez et al. 2023). POD places a financial burden on the healthcare system, increasing health care costs by approximately $114 billion per year for older adults (Leslie et al. 2008), and Medicare payments by $6.9 billion annually for delirium‐related in‐hospital complications (Inouye 2006). Proactive identification of risk factors prognostic of POD is important to provide an opportunity for early detection of at‐risk patients, help health professionals prevent delirium occurrence, and provide a foundation for future intervention development.

Known risk factors of POD after cardiac surgery are older age (Igwe et al. 2023; Wang et al. 2020), diabetes (Igwe et al. 2023), preoperative cognitive dysfunction (Igwe et al. 2023), and sleep disorders (Wang et al. 2020). Recently, the term ‘perioperative sleep disorders’ (PSD) has been used in the literature to describe sleep disorders either before or after surgery (Huang, Huang, et al. 2024), which are common in surgical patients (up to 60%; (Butris et al. 2023)). PSD can be defined as the presence of sleep breathing disorders (SBDs, such as sleep apnea), poor sleep quality or sleep deprivation, insomnia, hypersomnia, parasomnia, and sleep‐related movement disorders (Wang et al. 2020; Cok et al. 2019; Madsen et al. 2013; Rhon et al. 2019). Sleep disorders, such as insomnia and sleep‐related breathing disorders, are highly prevalent amongst patients with cardiovascular diseases (Zhang et al. 2024) and have been identified as significant predictors of adverse cardiovascular outcomes, including coronary artery disease (Kadier et al. 2022), which may require surgical intervention. Even irregular sleep duration and timing have been found to be significant risk factors for cardiovascular outcomes, independent of traditional cardiovascular disease risk factors (Huang et al. 2020). Also, a high percentage of patients with cardiovascular disease have undiagnosed sleep disorders (Shapira‐Daniels et al. 2020; Kirk et al. 2023; Suen et al. 2020) which could be diagnosed at the time of surgery.

The presence of PSD has been associated with an increased risk of short‐term postoperative complications (Lin et al. 2021; Rampes et al. 2019). Preoperative sleep duration and quality are linked to heightened postoperative pain (Roehrs and Roth 2017) and greater opioid consumption—both of which are established risk factors for the development of POD (Morrison et al. 2003; Gutierrez et al. 2024). Notably, pharmacologic interventions targeting sleep, such as zolpidem, have demonstrated multiple benefits in patients undergoing orthopaedic surgery (Gong et al. 2015). In addition to improving sleep efficiency and reducing pain intensity and opioid requirements, zolpidem has been associated with decreased postoperative nausea and vomiting and enhanced functional recovery, including improved range of motion (Gong et al. 2015). Poorer sleep (Kjølhede et al. 2012) was also independently predicted by increased opioid use, the need for antiemetics, postoperative weight gain, and reduced stress‐coping skills, all of which were linked to longer hospital stays and slower recovery (*p* = 0.002).

Although preventive interdisciplinary protocols such as the Hospital Elder Life Program (HELP) identify sleep deprivation as a major risk factor for POD incidence and encourage nocturnal sleep, sleep assessments are rarely provided pre‐operatively (Inouye et al. 1999; Huang, Wu, et al. 2024). PSD is one of the strongest risk factors for POD development (Wang et al. 2020; Huang, Huang, et al. 2024; He et al. 2022). Potential explanations for the co‐development of PSD and POD include brain–blood barrier disruption, circadian rhythm disturbance, inflammatory mediator activation due to anaesthesia and surgical stress, and oxidative stress (Wang et al. 2021). Prior research (He et al. 2022; Sun et al. 2022; Nagappa et al. 2017) has demonstrated that the presence of sleep‐related breathing disorders (SDBs) like obstructive sleep apnea (OSA; Sun et al. 2022; Nagappa et al. 2017) or sleep disturbances (defined as OSA and insomnia) (He et al. 2022) significantly increases the risk of POD. A systematic review (Fadayomi et al. 2018) found that the odds of developing POD after surgery are 5.2 times greater in individuals with sleep disturbances; more specifically, the odds of POD are 4.8 times greater in patients with OSA and 5.6 times greater in individuals with unspecified types of sleep disorders. However, these studies (He et al. 2022; Sun et al. 2022; Fadayomi et al. 2018) do not specifically focus on patients undergoing cardiac surgeries. Compared to patients undergoing noncardiac surgeries, those having cardiac surgeries (including coronary artery bypass grafting, heart transplant, aortic surgeries, arrhythmia and congenital heart defect surgeries) require deeper sedation (Brown 2014), cardiopulmonary pumping (O'Neal et al. 2017), and a longer duration of surgery (Greaves et al. 2020), all of which significantly increase the risk of POD and are mostly non‐modifiable (Varpaei, Farhadi, et al. 2024).

A synthesis of the literature on the association between sleep quality and POD before and after cardiac surgeries is lacking. Thus, the primary aim of our systematic review is to determine the association between PSD and POD in cardiac surgery patients during hospital stay. The secondary aim of our study is to explore the timing of return to baseline preoperative sleep quality amongst patients undergoing cardiac surgery between those with and without POD after hospital discharge.

## 2 Materials and Methods

### 2.1 Study Design

To address the aims, we conducted a systematic review and meta‐analysis. We utilised the Preferred Reporting Items for Systematic Reviews and Meta‐Analyses (PRISMA: see Supplements) (Page et al. 2021; Liberati et al. 2009) statement to guide the reporting of our findings. The protocol of this study was not published.

### 2.2 Eligibility Criteria

We considered articles to be eligible for inclusion if they evaluated the associations between sleep quality and POD amongst patients undergoing cardiac surgery. We included studies if they were:

1. Published in peer‐reviewed journal with available full text.
2. Original research, including study designs that evaluated the incidence of POD in relation to a comparison group (e.g., cross‐sectional studies, randomised controlled trials (RCTs), cohort and case–control studies).
3. Conducted with hospitalised adults > 18 years of age undergoing CS.

We excluded study protocols, editorials, letters to the editor, commentaries, conference abstracts, case reports, and case series, mindful that these documents lack a comparison group or data for analysis.

### 2.3 Search Strategy

**FIGURE 1:**
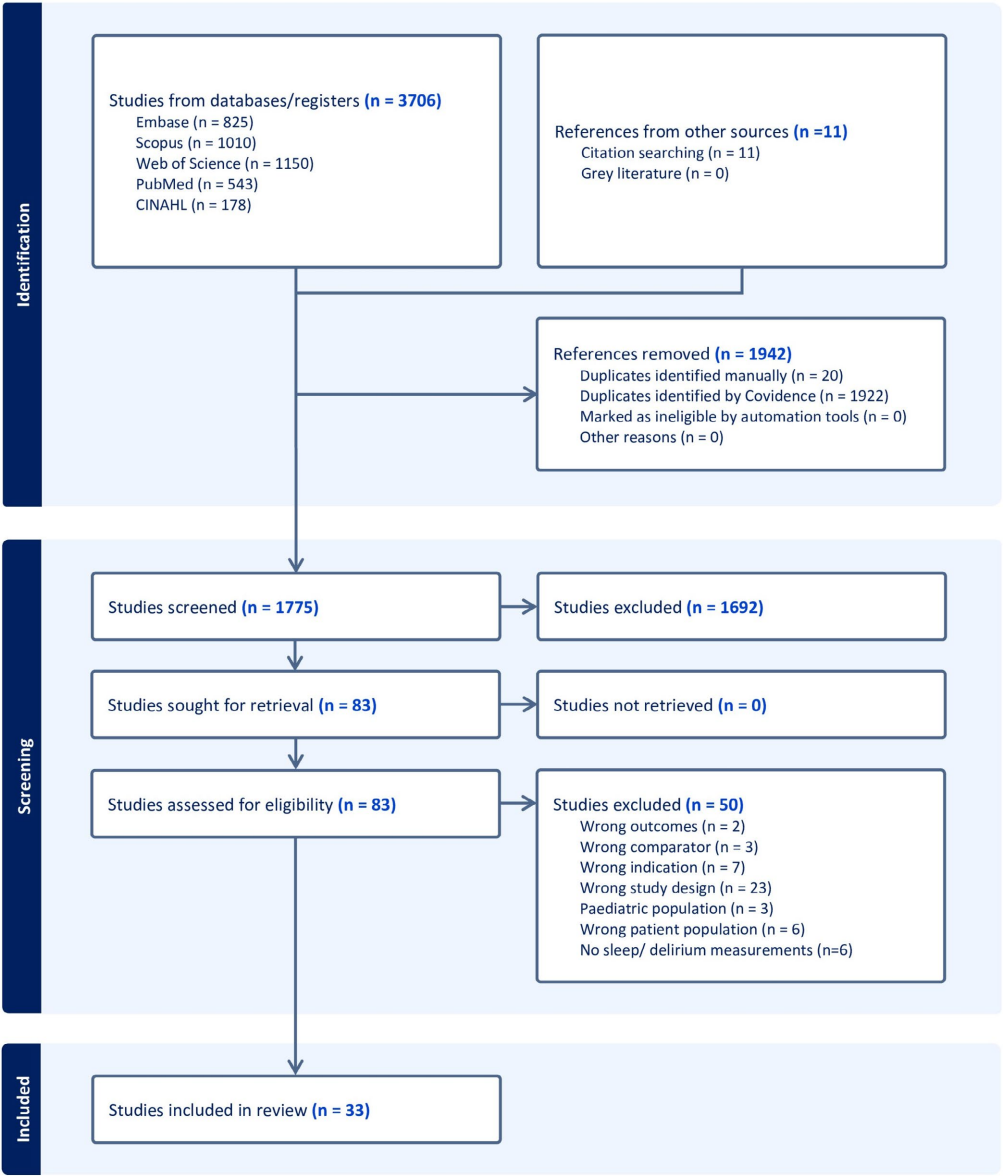
PRISMA flowchart of the systematic review.

We searched five electronic databases, PubMed (MEDLINE), Cumulative Index to Nursing and Allied Health Literature (CINAHL; EBSCO), Web of Science (WOS [Clarivate]), Scopus (Elsevier), and EMBASE. All relevant studies that originated from registries but were published and indexed in these databases were included and screened. The initial database search was conducted in July 2024, and it was updated in July 2025. To maximise sensitivity and inclusiveness, no search filters or restrictions (e.g., language, date, age, study design, or publication type, time of publication) were applied in any of the database searches. All database searches were conducted with the help and guidance of a trained and experienced master's‐prepared university librarian (Figure 1). All keywords and controlled vocabulary were modified for each database (Supplements). To identify potentially missed relevant articles, we employed (HV, KF) a snowball sampling technique by reviewing the discussion sections of the initially included articles, thereby capturing additional eligible studies referenced but not yet included in our review. We also conducted a hand search of reference lists for eligible studies (Table 1; Figure 1).

**TABLE 1:** Search terms and boolean operators used in online database search.

PubMed	((‘Delirium’ [Mesh] OR ‘postoperative delirium’ OR ‘post‐operative delirium’ OR delirium) AND (‘Sleep Initiation and Maintenance Disorders’ [Mesh] OR ‘Sleep’ [Mesh] OR ‘Sleep Disorders’ OR ‘Sleep Wake Disorders’ [Mesh] OR sleep* OR ‘sleep disturbance’ OR ‘sleep quality’) AND (‘Cardiovascular Surgical Procedures’ [Mesh] OR ‘Cardiac Surgical Procedures’ [Mesh] OR cardiac OR cardiovascular))
Web of science	TS = ((‘postoperative delirium’ OR ‘post‐operative delirium’ OR delirium) AND (sleep* OR ‘sleep disturbance’ OR ‘sleep quality’ OR ‘Sleep Wake Disorders’ OR ‘Insomnia’ OR ‘Sleep Pattern Disturbance’) AND (cardiac OR cardiovascular OR ‘cardiac surgery’ OR ‘cardiovascular surgery’ OR Cardiovascular Surgical Procedures))
Scopus	(‘postoperative delirium’ OR ‘post‐operative delirium’ OR delirium) AND (sleep* OR ‘sleep disturbance’ OR ‘sleep quality’ OR ‘sleep quality’ OR ‘insomnia’ OR ‘Sleep Wake Disorders’ OR ‘Sleep Pattern Disturbance’) AND (cardiac OR cardiovascular OR ‘cardiac surgery’ OR ‘cardiovascular surgery’ OR ‘Cardiovascular Surgical Procedures’)
Cumulated index to nursing and allied health literature	(MH ‘Delirium’ OR ‘post‐operative delirium’ OR ‘postoperative delirium’ OR delirium OR MH ‘Postoperative Period’) AND (sleep* OR ‘sleep disturbance’ OR sleep quality OR MH ‘Sleep Disorders’ OR MH ‘Insomnia’) AND (cardiac OR cardiovascular OR MH ‘Cardiac Surgery’ OR MH ‘Surgery, Cardiovascular’)
EMBASE	(sleep* OR ‘sleep disturbance’/exp. OR ‘sleep quality’ OR ‘sleep disorder’/exp. OR ‘sleep Wake Disorders’/exp. OR ‘insomnia’/exp) AND (‘cardiac surgery’/exp. OR ‘cardiac’/exp. OR cardiac OR ‘cardiovascular’/exp. OR cardiovascular OR ‘Cardiovascular Surgical Procedures’/exp) AND (‘postoperative delirium’/exp. OR ‘post‐operative delirium’ OR ‘postoperative delirium’ OR ‘delirium’/exp. OR delirium)

### 2.4 Exposures

Our exposure of interest was perioperative sleep disorders, with a particular focus on poor sleep quality, insomnia, history of sleep apnea such as OSA, and sleepiness during the daytime. For this review, sleep quality can either be assessed subjectively (patient‐reported surveys such as Pittsburgh Sleep Quality Index [PSQI]) or objectively by polysomnography (PSG) or via other wearable devices (e.g., actigraphy, Actiwatch). Sleep assessments could also be performed before and/or after surgery.

### 2.5 Outcomes

We elected to evaluate POD as the main outcome of the study. POD was measured by a valid assessment instrument (to avoid limiting the confusion assessment method [CAM; (Liberati et al. 2009; Inouye et al. 1990)], which is a standard assessment tool; the Delirium Rating Scale (Trzepacz et al. 2001); and others), clinician judgement, or diagnosis recorded in the patient's medical record. A full list of delirium assessment tools is provided in Supplements.

### 2.6 Study Selection

Two independent reviewers (HV and KF) independently screened titles, abstracts, and full texts using Covidence (Melbourne, Australia). During the screening process, discrepancies between reviewers were resolved through discussion and careful re‐evaluation of each study's alignment with the predefined inclusion criteria and research aims. If the content matched the research questions or aim, it was included; otherwise, it was excluded. Any disagreements between reviewers were resolved through discussion, with the involvement of a third‐party adjudicator (LR) when necessary (Cohen's Kappa title/abstract = 0.38, full text = 0.47).

### 2.7 Synthesis of Data

Using a pre‐designed data extraction form, two reviewers independently abstracted data from all included articles. Data were abstracted based on the study design; the country where the study was conducted; exposure and outcome measurement tools, including sleep and delirium assessment measures; participant characteristics (sex [% male], sample size, population, type of PSD); and results. Then, the data were synthesised narratively based on a methodological approach (study design; observational vs. interventional) and the time needed to return to preoperative sleep quality to address the concept of post‐operative sleep quality recovery. Any discrepancies in abstracted data between the two reviewers were resolved through discussion. The Standardised Mean Difference (SMD) is a statistical measure used in our meta‐analysis to compare differences in means across studies that may use different scales. For meta‐analysis purposes, we pooled data and applied the SMD to both subjective and objective sleep measures. The subjective sleep measure was the PSQI (continuous; mean and SD). Objective sleep measures were PSG variables, including total sleep time, apnea‐hypopnea index, sleep latency, and sleep efficiency. SMD represents the difference in means between patients with and without POD, standardised by the pooled standard deviation. A positive or negative SMD indicates whether poorer or better sleep characteristics are associated with a higher risk of POD. The magnitude of SMD follows conventional interpretations: 0.2 (small effect), 0.5 (moderate effect), and 0.8+ (large effect). In the context of this meta‐analysis, SMD is the mean difference in sleep measures (e.g., PSQI scores or PSG‐derived metrics) between patients with and without POD, adjusted by the pooled SD. To test for and quantify heterogeneity amongst studies, we used: (Whitlock et al. 2011) Cochran's *Q* test which assesses whether heterogeneity is greater than chance; (Evered et al. 2018) *I* ^2^ statistic which quantifies the percentage of total variation due to heterogeneity rather than sampling error. An *I* ^2^ of 0%–25% suggests low heterogeneity, 25%–50% suggests moderate heterogeneity, > 50% suggests substantial heterogeneity (Higgins et al. 2003); and (Chen et al. 2021) Tau^2^ (*τ* ^2^) which estimates the variance of effect sizes across studies. These methods help determine whether differences in study characteristics (e.g., population, measurement techniques) contribute to variability in the effect sizes.

We utilised R Studio (‘*meta*’ package (Schwarzer 2023)) to conduct a meta‐analysis of studies that provided sleep measures (objective or subjective) using random effects (due to high statistical heterogeneity of pooled data). Standardised Mean Differences (SMD) were used to report the effect sizes. If a study reported the median and IQR (Q3–Q1), we assumed the median was equal to the mean and calculated the standard deviation as the IQR divided by 1.35 (Higgins et al. 2023). We conducted subgroup analyses based on risk of bias (ROB). Studies were categorised as having low or moderate/high risk of bias using the Joanna Briggs Institute (JBI; 48) ROB tool. Meta‐analyses were stratified by ROB subgroup using the subgroup argument in the meta function from the R meta package (version 27771613, 2025‐02‐02). Pooled effect sizes and heterogeneity statistics were computed separately for each subgroup. We conducted a sensitivity analysis by removing a specific study to assess its influence on the overall effect size and determine whether its exclusion would significantly alter the meta‐analytic results (Supplements).

### 2.8 Risk of Bias Assessment

The JBI critical appraisal tools were used to conduct a risk of bias assessment (Barker et al. 2023). From the JBI list of critical appraisal tools, appropriate tools were selected for cross‐sectional, cohort, quasi‐experimental, and RCT studies. Criteria for risk of bias and quality assessment depend on the study design; but in general, the domains focus on the validity and reliability of exposure and outcome measurement, recruitment from a sample population, homogeneity of the population at baseline, randomisation and blinding (specifically for RCTs), and the appropriateness of statistical analysis. Two independent reviewers (HV and KF) independently evaluated articles. If any discrepancies occurred, the reviewers met and discussed them until reaching consensus regarding the final decision. Whilst no studies were excluded based on quality, we conducted a subgroup analysis in the meta‐analysis based on the risk of bias classification to explore its potential influence on outcomes (Supplements).

## 3 Results

### 3.1 Descriptives

**FIGURE 2:**
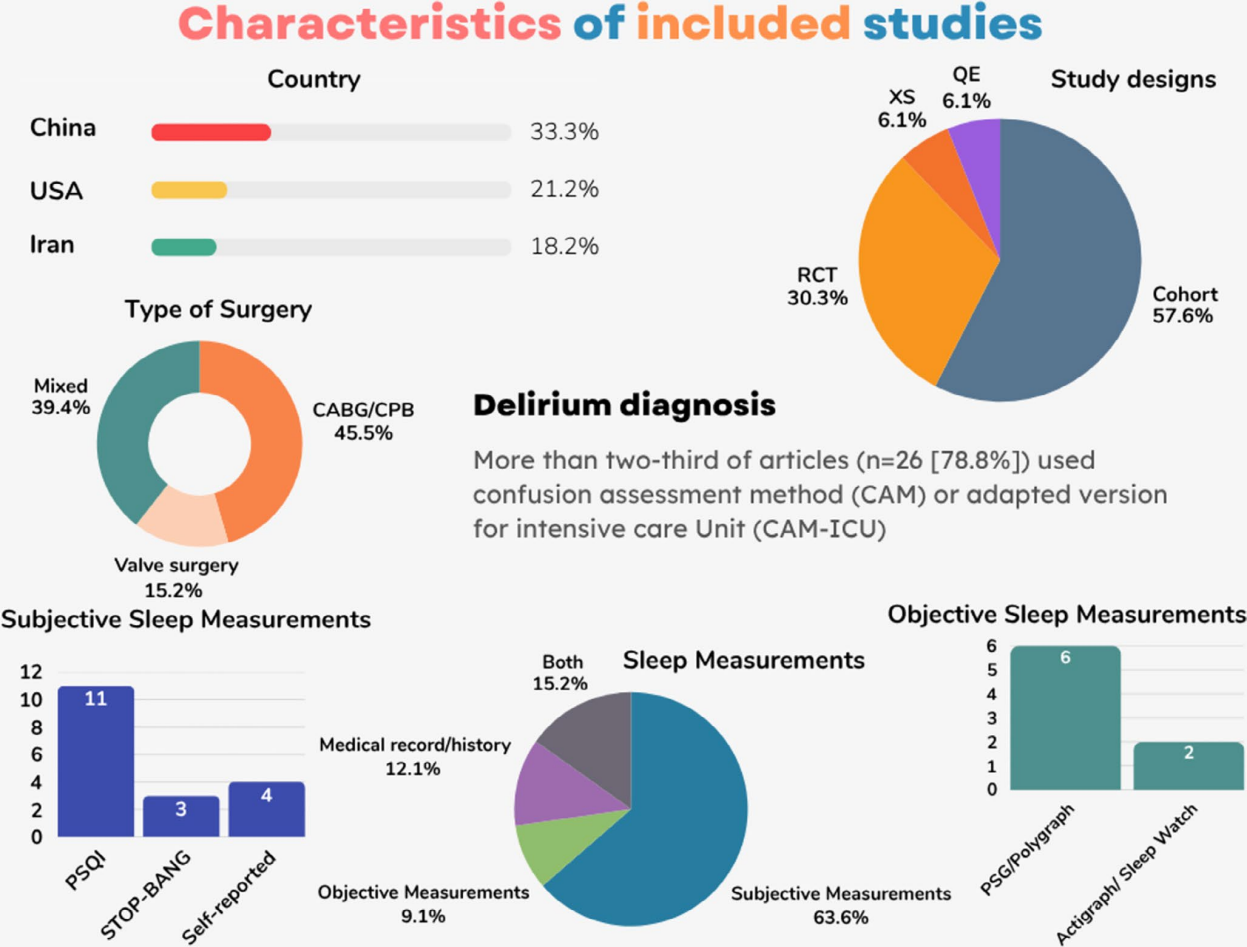
Characteristics of included studies, including country, study design, delirium assessment tools, and sleep measures. PSG = Polysomnography; PSQI = Pittsburgh Sleep Quality Index; QE = Quasi‐experimental; STOP‐BANG = Snoring, Tiredness, Observed apnea, high blood Pressure, Body mass index, Age, Neck circumference, and Gender; XS = Cross‐sectional.

A total of 3706 articles were identified from the five databases, and 11 additional articles were found from a citation search of the included studies. After removing 1942 duplicate citations (by Covidance), 1775 articles underwent title and abstract screening, of which 1692 articles were excluded. The remaining 83 articles underwent full‐text review. After excluding 50 full‐text articles, 33 remained for inclusion in this systematic review. Of the 33 included studies (de la Varga‐Martínez et al. 2023, 2021; Wang et al. 2020; Huang, Huang, et al. 2024; Huang, Wu, et al. 2024; Ibala et al. 2021; Qu et al. 2022; Roggenbach et al. 2014; Oldham et al. 2021; Zhang et al. 2015, 2017; Cheraghi et al. 2016; Dianatkhah et al. 2015; Atalan and Sevim 2013; Chen et al. 2020, 2024; Lin et al. 2023; Shorofi et al. 2024; Fazlollah et al. 2021; Huet et al. 2024; Javaherforooshzadeh et al. 2022; Turan et al. 2020; Tafelmeier et al. 2019; Rivas et al. 2023; Koster et al. 2009; Simeone et al. 2018; Freedman et al. 2025; Yang et al. 2025; Cai et al. 2025; Kim and Kim 2024; Fang et al. 2024; Mahmoudi et al. 2025), 63.6% were observational (19 cohort and two cross‐sectional), and 36.4% were interventional (10 RCTs and two quasi‐experimental). Most studies originated from China (33.3%, *n* = 11) followed by the USA (21.2%, *n* = 7) and Iran (18.2%, *n* = 6), with the remaining 9 conducted in other countries. Characteristics of eligible studies were provided in Table 2 and Figure 2.

**TABLE 2:** Characteristics of included studies.

Study (year)	Country	Sex (% male)	Sample size	Study design	Population	Type of PSD
Huang, Wu, et al. (2024)	China	37%	71	RCT	Valve replacement	Preop Sleep quality
Ibala et al. (2021)	USA	75%	16	Cohort	CPB	Preop Sleep quality
Wang et al. (2020)	China	56%	186	Cohort	On pump valve surgery	Preop Sleep quality
Roggenbach et al. (2014)	Germany	60%	92	Cohort	CABG, valve replacement	Sleep‐disordered breathing
Oldham et al. (2021)	USA	67%	15	Cohort	Aortic valve replacement	Preop insomnia, sleep quality, sleep‐disordered breathing
Zhang et al. (2015)	China	80%	249	Cohort	CABG	Postop sleep quality
Cheraghi et al. (2016)	Iran	60%	40	Cohort	Open heart (mostly CABG)	Postop sleep quality
de la Varga‐Martínez et al. (2023)	Spain	—	215	Cohort	Cardiac surgeries	Postop insomnia symptoms
Atalan and Sevim (2013)	Turkey	80%	51	Cohort	CABG with CPB	Postop sleep quality
Chen et al. (2020)	China	55%	20	Cohort	CPB	Postop sleep quality and architecture
Lin et al. (2023)	China	57%	55	Cohort	Valve surgery	Preop sleep quality and architecture
Javaherforooshzadeh et al. (2022)	Iran	65%	306	Cohort	Cardiac surgeries (mostly valve)	Sleep‐disordered breathing
Tafelmeier et al. (2019)	Germany	87%	141	Cohort	CABG	Sleep‐disordered breathing
Rivas et al. (2023)	USA	68%	590	Cohort	Cardiac surgeries	Sleep‐disordered breathing
Koster et al. (2009)	Netherlands	—	103	Cohort	Cardiac surgeries	Postop sleep disturbance
Huang, Huang, et al. (2024)	China	38%	194	Cohort	Cardiac surgeries	Preop Sleep quality
de la Varga‐Martínez et al. (2021)	Spain	78%	589	Cohort	CPB	Insomnia
Simeone et al. (2018)	Italy	84%	89	Cross‐sectional	Cardiac surgeries	Preop sleep disorder
Qu et al. (2022)	USA	73%	394	RCT	Cardiac surgeries	Pre/postop sleep disturbance
Dianatkhah et al. (2015)	Iran	76%	137	RCT	CABG	Pre/postop sleep quality
Shorofi et al. (2024)	Iran	68%	114	RCT	CABG	Postop sleep quality
Fazlollah et al. (2021)	Iran	43%	60	RCT	CABG	Postop sleep quality
Huet et al. (2024)	France	76%	331	RCT	Cardiac surgeries	Postop sleep quality
Turan et al. (2020)	USA	69%	794	RCT	CPB	Postop sleep quality
Zhang et al. (2017)	China	79%	278	Quasi experimental	CABG	Postop sleep difficulty
Freedman et al. (2025)	USA	75%	302	RCT	CBP	Preop sleep disturbance
Yang et al. (2025)	China	41%	76	RCT	CABG/CPB, valve	Pre/postop sleep quality
Cai et al. (2025)	China	79%	216	Cohort	Acute aortic dissection with CPB	Postop sleep quality
Chen et al. (2024)	USA	69%	4286	Cohort	CABG, valve, or aortic aneurism	Preop sleep‐disordered breathing
Kim and Kim (2024)	Korea	74%	195	Cohort	CABG, valve, or aortic aneurism	Postoperative sleep disorder
Lin et al. (2025)	China	61%	159	Quasi‐experimental	CABG or valve	Pre/postop sleep quality
Fang et al. (2024)	China	59%	100	RCT	Cardiac surgery	Preoperative sleep quality and insomnia
Mahmoudi et al. (2025)	Iran	67%	920	Cross‐section	CABG	Preop sleep disorder, postop sleep quality

Abbreviations: CABG, coronary artery bypass graft; CPB, cardiopulmonary bypass surgery (in table means cardiac surgery with CBP); RCT, randomised controlled trial.

Most studies assessed sleep (Table 3) preoperatively using validated instruments, with the Pittsburgh Sleep Quality Index (PSQI) being the most common subjective measure (11 studies), and polysomnography (PSG) the most frequent objective method (6 studies). Other tools included STOP‐Bang, Insomnia Severity Index, Richards Campbell Sleep Questionnaire, and Groningen or Leeds sleep scales.

The majority of patients underwent coronary artery bypass graft (CABG) or valve surgery, primarily amongst adults aged ≥ 18, though some focused on older adults (e.g., ≥ 60 years). Delirium incidence varied widely (3.6%–73%), reflecting heterogeneity in populations and assessment timing. Most studies used the Confusion Assessment Method (CAM or CAM‐ICU) to evaluate delirium, though the duration of assessment ranged from 2 to 7 days, and was often underreported or vague. Additional details are available in the Supplements.

### 3.2 Analysis and Synthesis of Evidence

#### 3.2.1 Observational Studies

Most studies that assessed subjective sleep quality using the PSQI before delirium assessment (Wang et al. 2020; Huang, Wu, et al. 2024; Ibala et al. 2021; Oldham et al. 2021; Cheraghi et al. 2016; Cai et al. 2025) and one study that measured the PSQI 12 months after the delirium diagnosis (Atalan and Sevim 2013) found a significant relationship (Table 1) between the incidence of POD and poor sleep quality (higher PSQI, higher incidence of POD). Studies that measured PSQI pre‐operatively also found this relationship, but the results were non‐significant (Ibala et al. 2021; Oldham et al. 2021). Although a higher PSQI score (> 5) was related to a higher incidence of POD, the two studies that found this relationship non‐significant restricted their study populations to older patients. Also, lower preoperative RCSQ (subjective measure of sleep quality) scores were not significantly related to the incidence of POD (Lin et al. 2023). However, preoperative self‐reported poor sleep quality (Zhang et al. 2015; Koster et al. 2009; Simeone et al. 2018) was significantly more prevalent in the POD group. Additionally, another study (de la Varga‐Martínez et al. 2023) found that patients who developed POD had a significantly higher prevalence of self‐reported sleep disturbances, nightmares, and difficulty falling and staying asleep, with these issues persisting up to 3 years after surgery. Oldham et al. (Oldham et al. 2021) reported that none of the subjective measures of PSD (i.e., PSQI, ISI, ESS, and STOP‐Bang) significantly predicted POD. Another study identified the presence of sleep‐disordered breathing (SDB) such as obstructive sleep apnea (OSA) as a significant risk factor for POD (Lin et al. 2023). Additionally, one study (Trzepacz et al. 2001) reported a lower incidence of POD in the high‐risk OSA group compared to low risk (2.3% vs. 6.1%), and another study (Rivas et al. 2023) found patients diagnosed with POD had more prevalent OSA compared to patients who did not experience POD (17% vs. 15%). In a large multicenter observational study, poor sleep quality (measured by PSQI) on the first postoperative night was a strong independent predictor of POD (OR = 9.08; 79).

Regarding the polysomnography findings, one study with older patients found higher total sleep time and sleep efficiency were significantly related to POD (Ibala et al. 2021), whilst a study on patients older than 18 found both total sleep time and sleep efficiency to be significantly lower in the POD groups (Lin et al. 2023). Two other studies showed that sleep time and sleep efficiency were reduced in POD patients, but the findings were not significant (Oldham et al. 2021; Chen et al. 2020). One study (Ibala et al. 2021) reported that sleep latency the day before surgery was significantly lower in POD patients, whilst another study (Oldham et al. 2021) found that sleep latency was higher 2–3 weeks before surgery in the POD group. Reduced rapid eye movement (REM; 52) before surgery was not a significant predictor of POD, but the percentage of REM stage one day post‐operatively was significantly lower in POD patients (Chen et al. 2020).

Pre‐operatively high AHI (as SBD indicator; > 27) and low oxygen saturation (< 91%) were also found to significantly predict the occurrence of POD (Roggenbach et al. 2014; Tafelmeier et al. 2019). In contrast, two studies (Oldham et al. 2021; Chen et al. 2020) found no significant polysomnographic findings related to predicting POD.

#### 3.2.2 Interventional Studies (RCTs and Quasi‐Experimental)

In terms of pharmacological interventions, Qu et al. (Qu et al. 2022) found that dexmedetomidine (anaesthetic) did not improve post‐operative sleep quality 30, 60, 90, and 180 days after surgery, as compared to a placebo, but significantly decreased the incidence of POD on day 1 post‐operatively. In elderly cardiac surgery patients with preoperative sleep disturbances (PSQI ≥ 8), short‐term intranasal dexmedetomidine significantly reduced the incidence of POD compared to placebo (12% vs. 30%, OR = 0.32; 78). In contrast, another study did not find a significant beneficial effect of dexmedetomidine on POD but showed better sleep quality with dexmedetomidine, when compared to placebo (Huet et al. 2024). Furthermore, another study (Turan et al. 2020) did not show that dexmedetomidine significantly reduced POD incidence. Although melatonin (3 mg 3 days before surgery until hospital discharge) improved post‐operative sleep quality when compared to oxazepam (10 mg; 55), the former medication had no significant preventive effect on POD (Dianatkhah et al. 2015). Administration of intranasal insulin (20 units) for 2 days pre‐operatively (last dose before anaesthesia induction) significantly decreased the incidence of POD, compared to placebo and furthermore, improved REM time, deep sleep, and sleep quality (Huang, Wu, et al. 2024). Another study supports these findings as administration of intranasal insulin (20 units) pre‐operatively and for two days postoperatively significantly reduced POD incidence and enhanced sleep efficiency and total sleep time in middle‐aged patients undergoing CPB surgery (Yang et al. 2025). Nonpharmacological intervention with earplugs and eye masks (after surgery in the intensive care unit) improved post‐operative sleep quality and reduced the incidence of POD (Shorofi et al. 2024). However, foot massage after cardiac surgery did not improve sleep quality (Fazlollah et al. 2021) or reduce POD. A before‐after intervention study implementing nursing protocols showed post‐operative sleep quality was a major risk factor for POD incidence, and the nursing intervention improved sleep quality (Zhang et al. 2017). Two quasi‐experimental studies (Zhang et al. 2017; Lin et al. 2025) demonstrated that nursing‐based interventions targeting modifiable risk factors effectively improved postoperative sleep quality and reduced the incidence of POD in cardiac surgery patients. Zhang et al. (Zhang et al. 2017) implemented a multicomponent nursing protocol (including pain control, orientation strategies, and minimising nighttime disruptions) and found that the intervention group had significantly better sleep quality ratings and a lower POD incidence (13.5% vs. 29.9%). Similarly, Lin et al. (Lin et al. 2025) reported that a comprehensive intervention program led to significantly higher sleep quality scores and lower POD incidence (24.7% vs. 47.6%) in elderly cardiac surgery patients.

### 3.3 Post‐Operative Sleep and Returning to Preoperative Sleep Quality

Patients who developed POD during hospitalisation had significantly higher post‐operative self‐reported sleep disturbances during ICU hospitalisation (Simeone et al. 2018) 1 year after surgery (Koster et al. 2009) and after 3 years (de la Varga‐Martínez et al. 2023). Similarly, a recent study using actigraphy found that postoperative sleep efficiency and duration improved over time but did not fully return to preoperative levels within the first postoperative week (Yang et al. 2025). Aligned with these findings, postoperative sleep disorder has been identified as a significant independent risk factor for POD (OR = 8.98) amongst cardiac surgery patients, even after transfer from ICU to the general ward (Kim and Kim 2024). It should be noted that the majority of studies that assessed long‐term sleep after hospital discharge used self‐reported (neither validated nor reliable) sleep disturbance measures (de la Varga‐Martínez et al. 2023; Koster et al. 2009; Simeone et al. 2018). Just two studies (Qu et al. 2022; Atalan and Sevim 2013) used valid and reliable subjective sleep measures and found that patients experiencing POD (Atalan and Sevim 2013) needed up to a month to return to their preoperative sleep quality but had a significantly higher PSQI score (> 5) 1 year after surgery (measured by PROMIS29; 50). Three and six months after surgery, the sleep quality score was lower than the preoperative sleep quality (indicating better quality sleep), which may indicate that full recovery of sleep quality may not be achieved until at least 3–6 months post‐surgery (Qu et al. 2022).

**TABLE 3:** Exposures and outcome measurements of included studies.

Study	Exposures		Outcome	Risk of bias
	Sleep measures			
	Subjective	Objective		
Huang, Wu, et al. (2024)	PSQI	Sleep monitoring watch	CAM‐ICU	Low
Ibala et al. (2021)	PSQI	PSG	CAM	Low
Wang et al. (2020)	PSQI	—	CAM‐ICU	Low
Roggenbach et al. (2014)	—	Portable polygraphs	CAM‐ICU	Moderate
Oldham et al. (2021)	PSQI, ISI, ESS, STOP‐Bang	PSG	3D‐CAM, DRS	Moderate
Zhang et al. (2015)	Self‐reported sleep quality	—	CAM‐ICU	High
Cheraghi et al. (2016)	PSQI	—	CAM‐ICU	High
de la Varga‐Martínez et al. (2023)	Self‐reported insomnia symptoms	—	CAM‐ICU	Moderate
Atalan and Sevim (2013)	PSQI	—	DSM‐IV, CAM‐ICU	Moderate
Chen et al. (2020)	—	PSG	CAM‐ICU	Moderate
Lin et al. (2023)	RCSQ	PSG	CAM‐ICU	Low
Javaherforooshzadeh et al. (2022)	STOP‐Bang	—	Clinician diagnosis/medical record	Moderate
Tafelmeier et al. (2019)	—	Portable SDB monitor	CAM‐ICU	Low
Rivas et al. (2023)	STOP‐Bang	—	CAM‐ICU	Low
Koster et al. (2009)	Self‐reported sleep disturbance	—	DSM‐IV	High
Huang, Huang, et al. (2024)	PSQI	—	CAM/CAM‐ICU	High
de la Varga‐Martínez et al. (2021)	Medical history/record of insomnia	—	CAM‐ICU	High
Simeone et al. (2018)	Self‐reported sleep disorder	—	CAM‐ICU	Low
Qu et al. (2022)	PROMIS	—	CAM	Moderate
Dianatkhah et al. (2015)	GSQS	—	Clinician diagnosis	Moderate
Shorofi et al. (2024)	VSHSS	—	NEECHAM	Moderate
Fazlollah et al. (2021)	RCSQ	—	DOS	Moderate
Huet et al. (2024)	Sleep quality numerical scale, LSEQ	—	CAM‐ICU	Low
Turan et al. (2020)	Sleep Interference scale	—	CAM‐ICU	Low
Zhang et al. (2017)	Clinician monitoring sleeping difficulties/Medical record	—	CAM‐ICU, DRS‐R‐98	Low
Freedman et al. (2025)	PROMIS	—	CAM	Low
Yang et al. (2025)	PSQI, ISI	Actigraphy	CAM‐ICU	Low
Cai et al. (2025)	PSQI	—	CAM/CAM‐ICU	Low
Chen et al. (2024)	Medical history/record of OSA	—	CAM	Moderate
Kim and Kim (2024)	Medical history/record of OSA	—	Nu‐DESC	Moderate
Lin et al. (2025)	VAS/RCSQ	—	3‐D CAM/CAM‐ICU	Low
Fang et al. (2024)	PSQI	—	CAM/CAM‐ICU	Low
Mahmoudi et al. (2025)	PSQI	—	CAM‐ICU	Low

Abbreviations: CAM, confusion assessment method; CABG, coronary artery bypass grafting; CPB, cardiopulmonary bypass; DRS, delirium rating scale; DOS, delirium observational screening scale; ESS, epworth sleepiness scale; GSQS, Groningen sleep quality score; HRV, heart rate variability; LSEQ, leeds sleep evaluation questionnaire; NEECHAM, Neelon and Champagne confusion scale; OSA, obstructive sleep apnea; PSQI, Pittsburg sleep quality index; PROMIS, patient‐reported outcomes measurement information system; RCT, randomised controlled trial; RCSQ, Richards Campbell sleep questionnaire; VAS, visual analogue scale; VSHSS, Verran and Snyder‐Halpern Sleep Scale.

### 3.4 Meta Analysis

**FIGURE 3:**
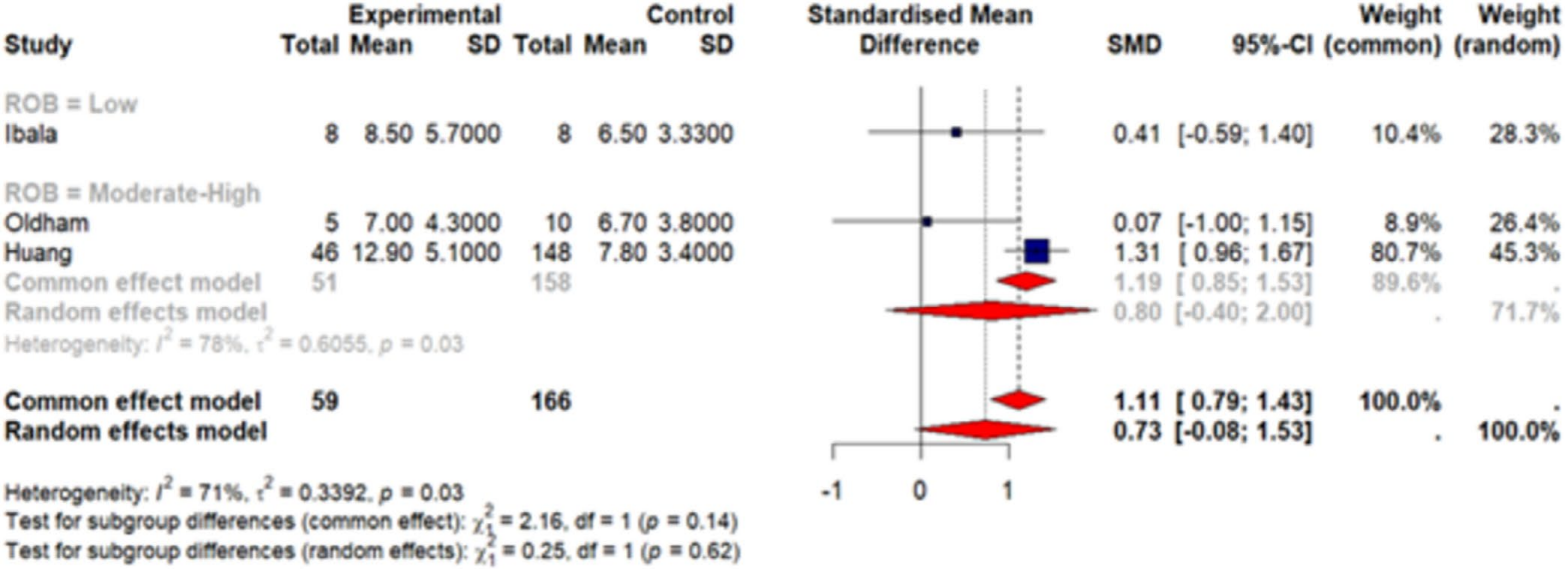
Forest plot of meta‐analysis on the association of Pittsburgh sleep quality index (PSQI) with post‐operative delirium (POD) in cardiac surgery patients (experiment = patients who developed POD).

**FIGURE 4:**
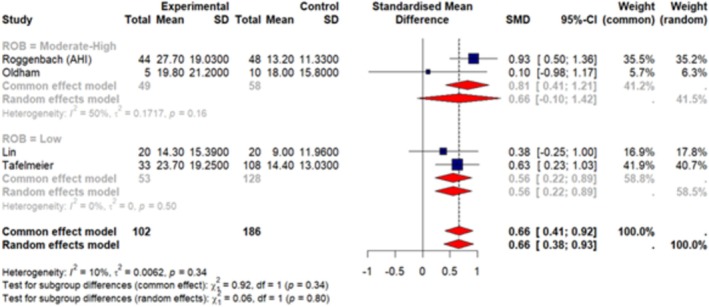
Forest plot of meta‐analysis on the association of apnea‐hypopnea index (AHI) with POD in cardiac surgery patients.

**FIGURE 5:**
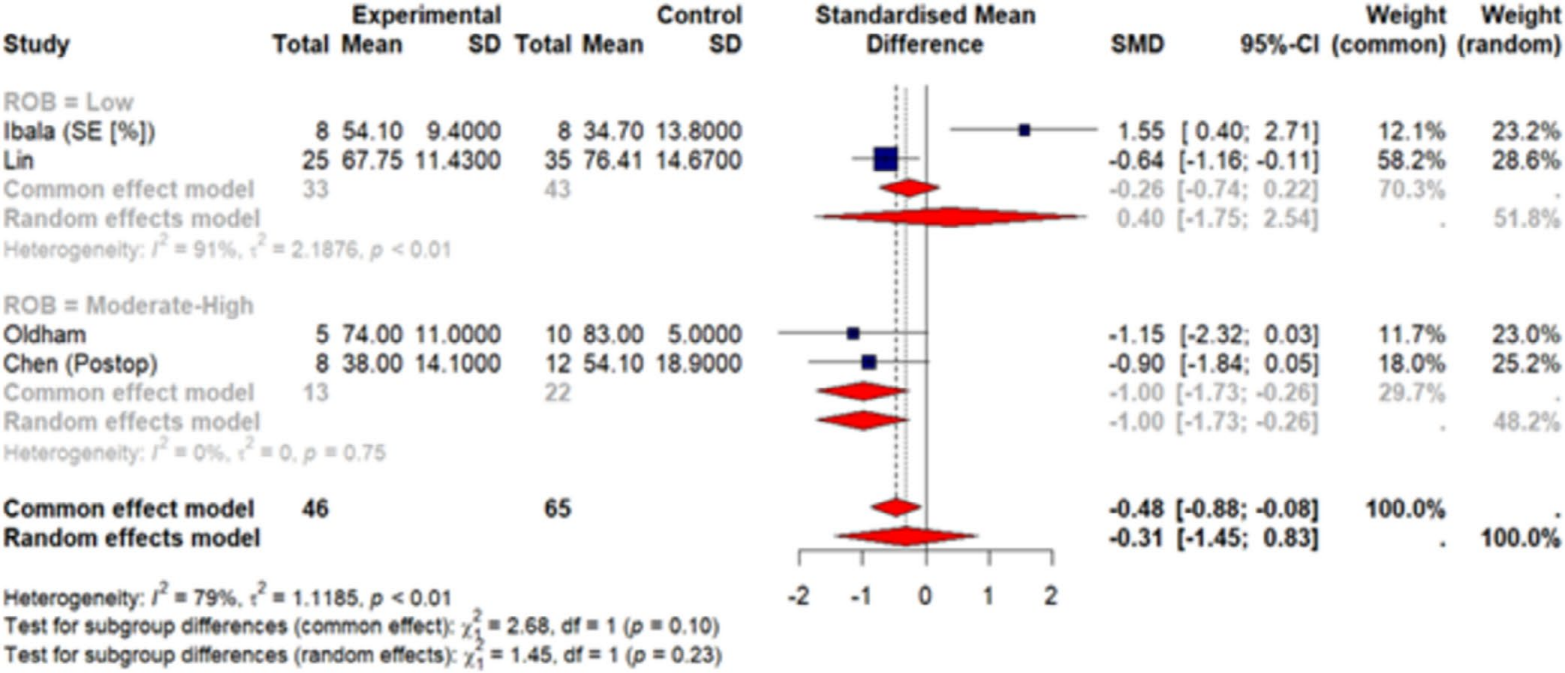
Forest plot of meta‐analysis on the association of sleep efficiency (SE [%]; measured by PSG) with POD in cardiac surgery patients.

**FIGURE 6:**
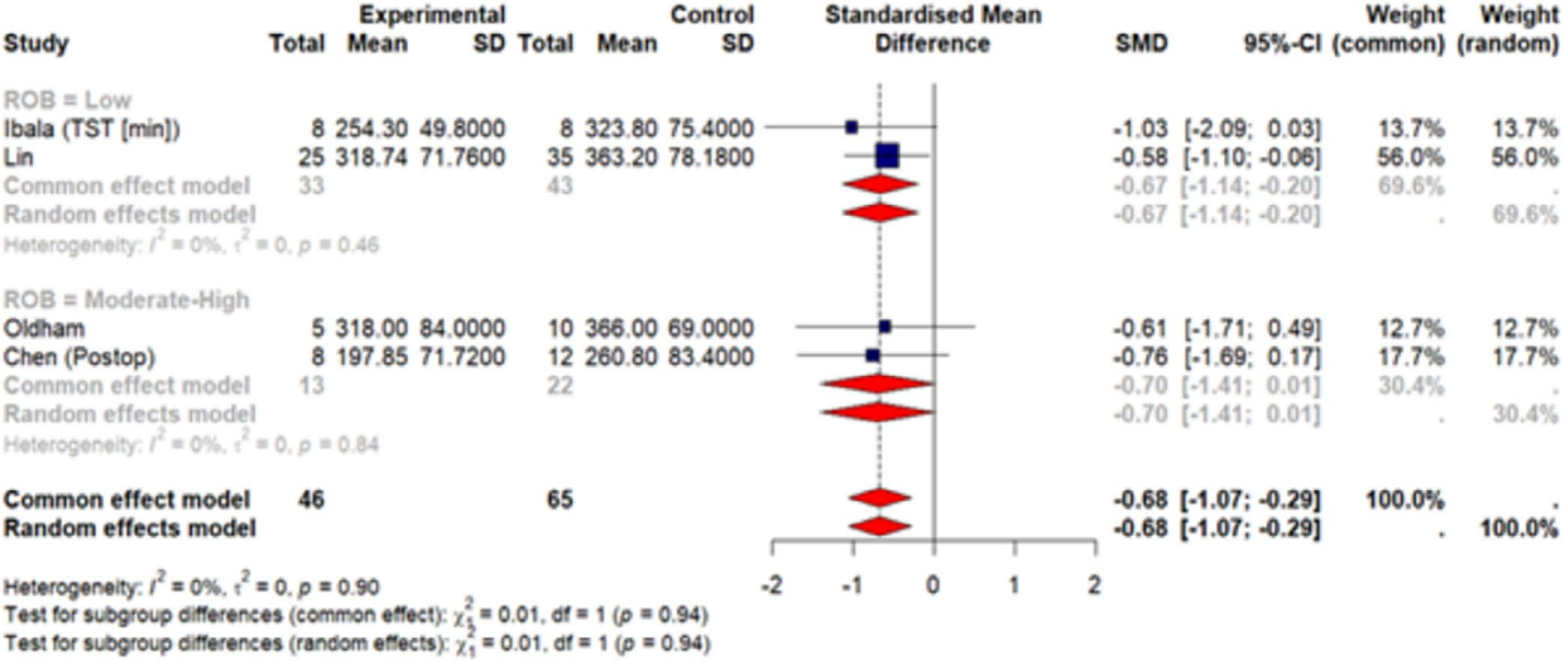
Forest plot of meta‐analysis on the association of total sleep time (TST [min]; measured by PSG) with POD in cardiac surgery patients.

**FIGURE 7:**
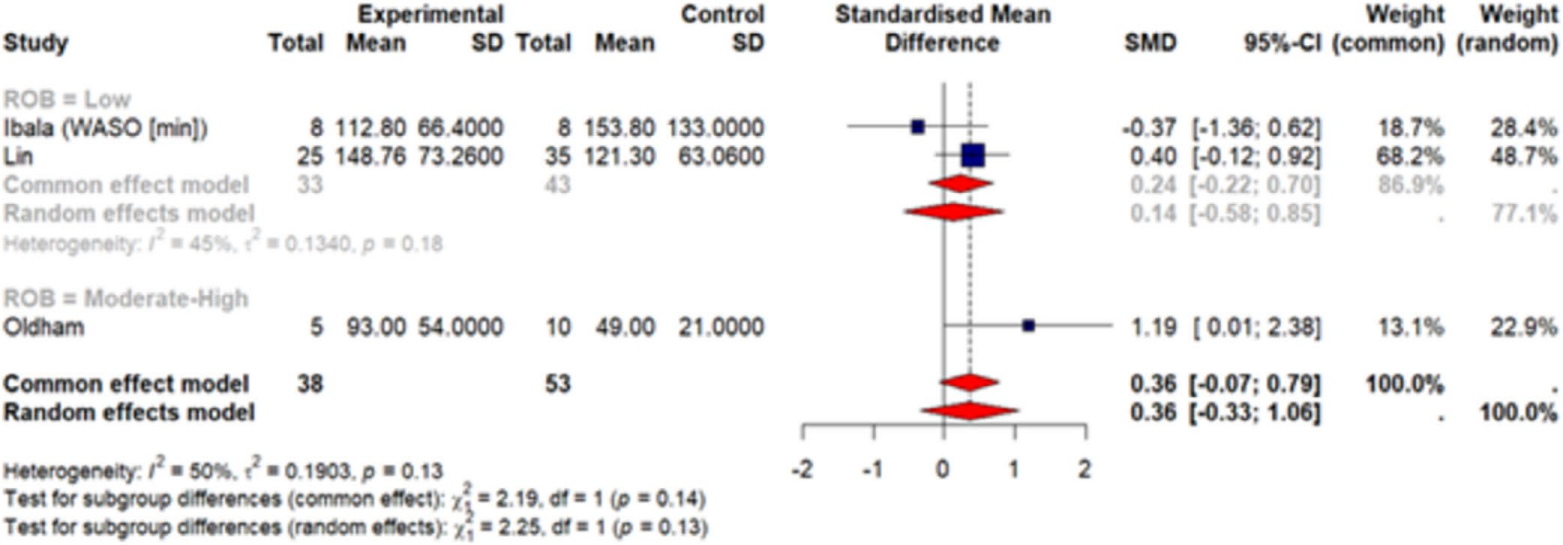
Forest plot of meta‐analysis on the association of wakefulness after sleep onset (WASO [min]; measured by PSG) with POD in cardiac surgery patients.

A total of 7 articles (Huang, Wu, et al. 2024; Ibala et al. 2021; Roggenbach et al. 2014; Oldham et al. 2021; Chen et al. 2020; Lin et al. 2023; Turan et al. 2020) were included in our meta‐analysis. These studies reported sleep measures as mean ± SD so we were able to pool data together. Our results (Figures 3, 4, 5, 6, 7) show a significant association between PSQI and POD occurrence, with a SMD of 0.73 (random effect 95% CI: −0.08–1.53; Figure 2), indicating higher PSQI scores are associated with a greater likelihood of POD occurrence. Similarly, increased AHI (random effect SMD = 0.66, 95% CI: 0.41–0.92; Figure 3) and WASO (random effect SMD = 0.36, 95% CI: −0.07–0.79; Figure 6) were associated with an increased likelihood of POD occurrence. Lower sleep efficiency (random effect SMD = −0.31, 95% CI: −1.45–0.83), sleep latency (random effect SMD = −0.86, 95% CI: −3.56–1.85), and total sleep time (random effect SMD = −0.68, 95% CI: −1.07 to −0.29; Figure 5) were associated with an increased risk of POD. None of the tests for subgroup analysis differences were significant; however, we recommend caution in interpreting these results because the findings may be due to the reduced sample sizes that occur when subgroup differences are examined (i.e., type 2 error).

Significant heterogeneity was observed in the meta‐analyses of SL (*I* ^2^ = 90%, *p* < 0.01), SE (*I* ^2^ = 79%, *p* < 0.01), and the PSQI (*I* ^2^ = 71%, *p* = 0.03) in relation to POD. The high *I* ^2^ values indicate considerable variation across studies, suggesting that factors beyond random error may contribute to these differences. Given this high heterogeneity, caution is needed when interpreting these results. Also, the sensitivity analyses did not yield any significant differences in the overall effect size or direction of the results.

### 3.5 Risk of Bias Assessment

**FIGURE 8:**
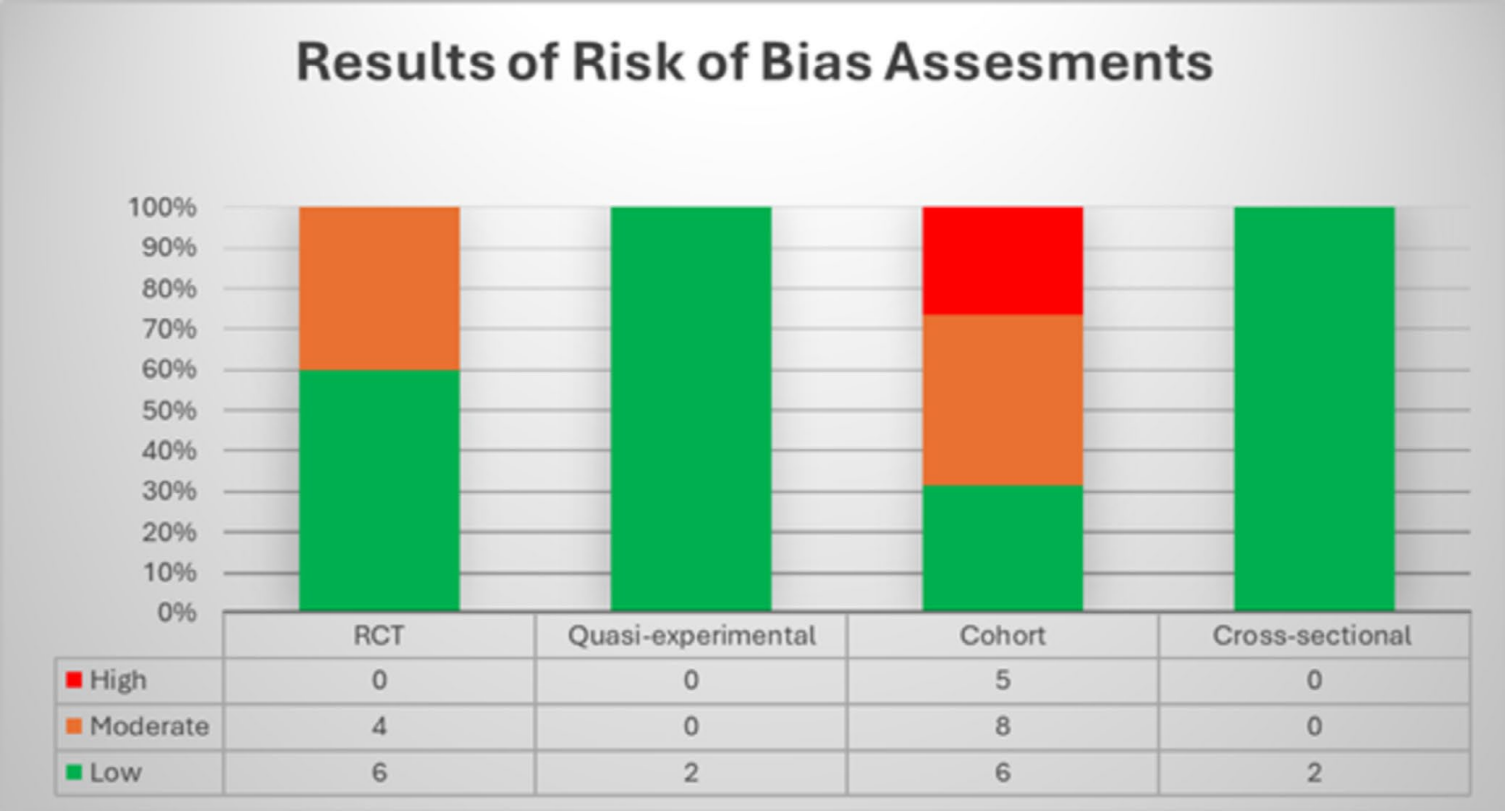
Distribution of risk of bias assessment across study designs.

Forty percent of all articles were identified as having a low risk of bias (Table 2 and Figure 8). For the RCTs and cohort studies, 40% and 31.57%, respectively, had a low risk of bias; for both cross‐sectional and quasi‐experimental studies, 100% had low risk of bias. In cohort studies, reasons for a high risk of bias include differences in baseline characteristics, issues with the validity and reliability of exposure measurements, challenges in identifying confounders and applying appropriate statistical approaches to address them, insufficient follow‐up time for the outcome to occur, and strategies to address incomplete follow‐up. One quasi‐experimental study and one cross‐sectional study were both determined to have a low risk of bias. The table containing details of the risk of bias assessment is provided in Supplements.

## 4 Discussion

Our primary finding confirms that PSD (including insomnia, poor sleep quality, and sleep‐disordered breathing) is significantly associated with an increased risk of POD in cardiac surgery patients. This association was evident across both subjective and objective sleep measures. Specifically, our meta‐analysis results showed that worse subjective sleep quality scores and objective sleep measurements, such as an increased apnea‐hypopnea index and decreased total sleep time, are significant risk factors for POD after cardiac surgery. These findings align with previous systematic reviews across general surgical populations, which reported that PSD is a modifiable risk factor for POD (OR = 3.73–5.24) (He et al. 2022; Fadayomi et al. 2018). Notably, our sensitivity and subgroup analyses based on risk of bias confirmed the robustness of these associations, underscoring the clinical relevance of addressing PSD in this high‐risk population. The pathophysiological link between PSD and POD may involve shared neuroinflammatory pathways, disrupted circadian regulation, and impaired melatonin secretion—mechanisms which are particularly relevant in cardiac surgery due to cardiopulmonary bypass and systemic inflammation (Wang et al. 2021). These biological pathways may help explain why patients with PSD are more vulnerable to developing POD in the immediate postoperative period (Pang et al. 2022).

Regarding our secondary aim, we observed that patients may require at least one month to return to preoperative sleep quality; however, the time needed varies depending on individual baseline status, as many patients already had poor sleep prior to surgery (PSQI > 5). Sleep disturbances may persist well beyond the acute postoperative period, with one study reporting effects lasting up to 3 years (de la Varga‐Martínez et al. 2023). More consistently, studies indicate that full sleep recovery often occurs between 3 and 6 months postoperatively (Qu et al. 2022; Bakry et al. 2022). These findings suggest that sleep is both an acute and long‐term postoperative concern that warrants clinical attention. Currently, there are few studies (Bakry et al. 2022; Hu et al. 2022) that report post‐operative sleep quality following CS, and the time needed to return to preoperative sleep quality. One study (Bakry et al. 2022) from Egypt, which used a self‐reported questionnaire, found that about 41% of patients needed more than 3 weeks to restore their sleep quality. Additionally, almost 40% of patients reported both difficulty falling asleep and waking up early in the morning, which are typical symptoms of insomnia. The study identified several reasons for poor sleep quality following CS, including pain at the wound site (reported by roughly one‐third of patients), followed by feelings of being hot, severe cough, and fatigue. A study from China (Hu et al. 2022) reported that almost half of the patients, 2 weeks after CS, experienced poor sleep quality (PSQI > 8), and roughly one‐third experienced daytime sleepiness (ESS > 9). Although sleep quality did not change 6 months after CS in the same group of patients (43% with PSQI > 8), daytime sleepiness decreased (13%). The study assessed sleep objectively (using actigraphy at 2 weeks post‐CS) and found that total sleep time and sleep efficiency significantly improved from week 1 to week 2 post‐operatively, whilst sleep latency and awakening frequency significantly decreased. Age, sleep efficiency, PSQI, total sleep time, and the duration of awakenings during the hospital stay were significant risk factors for sleep quality 6 months after CS. Essentially, based on these findings along with our own, it seems that insomnia and poor sleep quality are the most common post‐CS sleep disorders, especially after hospital discharge, and these issues are primarily due to pain and feeling hot. Yet, there is a lack of knowledge about potential interventions to improve sleep quality and insomnia post‐operatively, either during hospitalisation or after discharge, and more randomised controlled trials are warranted.

Our findings highlight the prevalence and potential impact of PSD. Whilst addressing sleep disorders prior to surgery may be important, it is often not feasible, particularly in urgent or emergent cases, due to time constraints, preoperative stress, and patient anxiety. These factors may limit the implementation of effective preoperative sleep interventions. Furthermore, the surgery itself and anaesthetic agents (e.g., opioids) can alter sleep architecture postoperatively by reducing slow‐wave sleep and suppressing REM sleep and arousals (Dimsdale et al. 2007; Shaw et al. 2005), compounding the risk of PSD. In addition, trauma caused by major surgeries, such as CS, triggers an innate and adaptive immune system resulting in an inflammatory response (Dąbrowska and Słotwiński 2014; Marik and Flemmer 2012; Alam et al. 2018). The inflammatory response leads to neuroinflammation (Alam et al. 2018), which is thought to cause postoperative sleep disturbances (Alam et al. 2018; Lin et al. 2000; Vgontzas et al. 1999) and POD (Pang et al. 2022; Brattinga et al. 2022; Hála 2007). Therefore, it appears that an inflammatory response (increased C‐reactive protein [CRP] and interleukin‐6 [IL‐6]) mediates the pathophysiology of POD (Liu et al. 2018). Additionally, preoperative CRP levels, along with the PSQI, have been shown to be elevated in patients who experienced POD after non‐CS procedures (Zheng et al. 2023). Given that OSA patients have elevated levels of IL‐6 (Imani et al. 2020; Fernandes et al. 2024) and CRP (Huet et al. 2024; Shamsuzzaman et al. 2002), it is important to recognise that patients with PSD may be at increased risk. Knowing this risk factor can help healthcare professionals not only consider close monitoring for POD after surgery but also anticipate that patients may continue to experience sleep disturbances as an unpleasant symptom even several months postoperatively.

From interventional studies (Qu et al. 2022; Dianatkhah et al. 2015; Shorofi et al. 2024; Fazlollah et al. 2021; Huet et al. 2024; Turan et al. 2020; Freedman et al. 2025; Yang et al. 2025; Fang et al. 2024) included in this review, the majority included pharmacological interventions (Qu et al. 2022; Dianatkhah et al. 2015; Huet et al. 2024; Turan et al. 2020; Freedman et al. 2025; Yang et al. 2025; Fang et al. 2024), and the others were nonpharmacological interventions (Shorofi et al. 2024; Fazlollah et al. 2021). Based on the findings of this review and other studies, melatonin (compared to oxazepam) could improve sleep quality after CS, but no significant difference in POD was observed (Dianatkhah et al. 2015); and dexmedetomidine prevented POD but had no significant impact on post‐operative sleep quality (Qu et al. 2022; Fang et al. 2024). Previous systematic reviews (Barnes et al. 2023; Shin et al. 2024) confirmed that melatonin can significantly reduce the incidence of POD in major surgeries (OR = 0.41 (Vgontzas et al. 1999); risk ratio = 0.57 (Shin et al. 2024)). Two other studies (Huet et al. 2024; Turan et al. 2020) did not support that dexmedetomidine is beneficial for POD prevention. All the latter studies (Qu et al. 2022; Huet et al. 2024; Turan et al. 2020; Fang et al. 2024) used the same POD assessment tool (CAM/ICU), but the others (Qu et al. 2022; Turan et al. 2020) focused on people older than 60 and one (Huet et al. 2024) focused on a wide range of people (18–85 years old). Possibly, differences in the findings could be due to the different doses and timing of dexmedetomidine administration. Qu et al. (Qu et al. 2022) started dexmedetomidine after surgery (post‐extubation) with a dose of 1 μg/kg/40 min, followed by 3 consecutive nights of administration. In contrast, Turan et al. (Turan et al. 2020) started dexmedetomidine in the operating room with a dose of 0.1 μg/kg/h, which was then increased to 0.2 μg/kg/h and later to 0.4 μg/kg/h post‐operatively for 24 h. Huet et al. (2024) administered a range of infusion doses of dexmedetomidine (0.1–0.4 μg/kg/h) post‐operatively, continuing until ICU discharge or for 7 days. Based on the significant findings of Qu et al. (2022), post‐operative administration (infusion) of dexmedetomidine for just 3 days may help prevent POD. Also, the other two studies (Maagaard et al. 2023; Duan et al. 2018) showed that administration of dexmedetomidine can prevent POD. The suggested dexmedetomidine loading dose was 0.4–0.6 μg/kg, and the infusion rate was 0.1–0.5 μg/kg (Duan et al. 2018). Melatonin and dexmedetomidine are not typically included as first‐line treatments in delirium or insomnia management guidelines, but are sometimes used in specific situations, with melatonin primarily for sleep‐promoting effects in older adults or those with circadian rhythm disturbances, and dexmedetomidine for sedation in critically ill patients or those requiring light sedation (Trzepacz et al. 1999). In addition to these agents, intranasal insulin has emerged as a promising pharmacological intervention for perioperative neuroprotection (Huang, Wu, et al. 2024; Yang et al. 2025). Two randomised controlled trials (Huang, Wu, et al. 2024; Yang et al. 2025) demonstrated that administration of intranasal insulin (20 units) either preoperatively alone or both pre‐ and postoperatively significantly reduced the incidence of POD and improved sleep parameters such as REM sleep, deep sleep, sleep efficiency, and total sleep time. These effects were particularly pronounced in middle‐aged patients undergoing CPB surgery (Yang et al. 2025), which may imply a potentially age‐ and context‐specific benefit of intranasal insulin through modulation of the sleep–wake cycle.

Findings from the nonpharmacological interventions were aligned with results from other studies, showing that wearing earplugs and an eye mask at night significantly improved sleep quality and reduced the incidence of POD (Shorofi et al. 2024), but foot reflexology massage did not show any significant impact on sleep or POD (Fazlollah et al. 2021). Furthermore, two quasi‐experimental studies (Zhang et al. 2017; Lin et al. 2025) supported that nursing‐led multicomponent or comprehensive interventions not only reduced POD incidence but also significantly improved postoperative sleep quality. These findings underscore the potential of nonpharmacological, nursing‐based strategies for optimising perioperative sleep to mitigate delirium risk after cardiac surgery. To improve in‐hospital post‐operative sleep, some nonpharmacological interventions such as music therapy, cognitive behaviour therapy, and eye masks and earplugs at night could significantly improve sleep quality after CS (Soh et al. 2024). Finally, in our previous research (Varpaei, Robbins, et al. 2024), we found that, although anaesthesia practise and management are essential to prevent post‐operative cognitive dysfunction after cardiothoracic surgeries, cognitive dysfunction after cardiothoracic surgeries is a multifactor phenomenon (Varpaei, Farhadi, et al. 2024) and requires a holistic approach to evaluate the condition and intervene. Preventing POD is also critical, as POD is a known risk factor for long‐term cognitive decline (Varpaei, Farhadi, et al. 2024). These findings highlight the importance of developing nursing‐led interventions for delirium prevention, as nurses have continuous and direct interaction with patients during the postoperative period. Compared to multidisciplinary programs such as the HELP, which, although effective, can be resource‐ and time‐intensive, nursing‐driven approaches may offer a more feasible and cost‐effective strategy for routine implementation in clinical settings (Inouye et al. 1999).

It is important to recognise that age is a significant factor in both sleep and POD. It is well known that older age is a significant non‐modifiable risk factor of POD (Sadeghirad et al. 2023). Additionally, age‐related sleep changes occur (Boulos et al. 2019). Every 10 years of age (beyond 18) is linked to a decrease of 10 min in total sleep time, a decrease of 2.1% in sleep efficiency, an increase of 9.7 min in WASO, an increase of 1.1 min in sleep latency, an increase of 2.1 events per hour in the arousal index, and an increase of 1.2 events per hour in the AHI (Boulos et al. 2019). All studies in this review included adult patients older than 18, but the majority included adults older than 60 (Boulos et al. 2019). Although age is a non‐modifiable risk factor, it should be considered when developing tailored interventions in future studies.

Although in this paper we used the term ‘poor sleep quality’ or PSD, it should be noted that this term is very broad. In future studies, researchers may need to consider specifying sleep disturbance (e.g., low sleep quality, insomnia, or low sleep efficiency) for clarity and to enhance understanding of sleep trajectory from before and after surgery.

### 4.1 Strengths and Limitations

This review offers an extensive overview of published research on sleep and POD in CS patients. The search strategy was systematic and could be replicated. However, grey literature searches were not conducted; we made this decision because we found multiple high‐quality peer‐reviewed studies evaluating the phenomena of interest. Although we developed a broad, multi‐database search strategy in consultation with a university health sciences librarian, it is possible that some relevant studies were not retrieved due to indexing inconsistencies or variable terminology across databases. However, we revised our original search terms and strategy to avoid introducing bias, whilst maintaining the inclusivity and comprehensiveness of our initial approach. Also, citation tracking was used to enhance completeness without compromising methodological integrity. The inter‐rater agreement during title and abstract screening was modest (*κ* = 0.38), reflecting challenges in early‐stage interpretation. However, agreement improved at the full‐text stage (*κ* = 0.47) following protocol refinement and team calibration. It should be noted that there are very few interventional studies on treatments for PSD to prevent POD in this population, especially in the post‐surgery period, given that the results show patients continue to struggle with their sleep disorders after surgery. Additionally, there was little uniformity in the indices used to describe sleep, and the definition of PSD was also very broad. Finally, due to the very limited number of studies included in the meta‐analysis, the quantitative results are preliminary findings, and their interpretation requires caution.

## 5 Implication for Research

Based on our findings, we suggest using valid and reliable tools (e.g., ESS, ISI, STOP‐Bang, and PSQI) when assessing sleep subjectively. When possible, objective measures rather than patient self‐report should be employed. Post‐operative sleep is not yet well investigated, and long‐term follow‐up of post‐operative sleep (9 months, 1 year, and so on) is needed to establish a foundation for the development of tailored interventions to accelerate sleep quality recovery so that patients can have a normal sleep or at least achieve increased preoperative sleep quality. Additionally, more interventional studies are needed to enhance post‐operative sleep quality and manage insomnia, specifically targeting environmental stressors such as temperature, noise, and light, as well as managing discomfort, particularly wound pain and cough. Conducting a concept analysis is strongly suggested to define the concept of ‘sleep recovery’ in the context of surgical patients.

### 5.1 Implications for Practice

Based on the findings of this review, we suggest that all patients who are candidates for major surgeries (e.g., cardiac, transplant, orthopaedic, neurological, pulmonary, and abdominal surgeries) undergo a sleep quality assessment using standard tools such as PSQI. Although polysomnography a few days before surgery might not be practical, it is suggested that patients scheduled for CS (i.e., a month before surgery) who have risk factors for OSA (obesity [BMI > 30]) or who complain about their sleep undergo polysomnography, if resources and insurance are available to cover the equipment and costs. Whilst polysomnography is still the gold standard for objective sleep assessment, it is resource‐intensive and expensive. As a result, actigraphy offers a useful option for measuring sleep patterns in clinical and research contexts. Any abnormal sleep quality findings or any PSD indicator such as a high (> 5) PSQI score or history of sleep disorders (e.g., OSA) need to be considered as a serious risk factor for POD, and patients need to be closely monitored after surgery for POD. Some institutions may have different preemptive protocols for POD prevention such as the Minimising ICU Neurological Dysfunction with Dexmedetomidine‐induced Sleep (MINDDS) protocol (Inouye et al. 1999). Patients' sleep complaints such as symptoms of insomnia (e.g., difficulty falling asleep, difficulty maintaining sleep) should be noted and recorded properly for future clinical follow‐up. Interventions such as reducing noise and light post‐operatively are warranted to ensure good sleep quality, both during the hospital stay and after hospital discharge. Appropriate pain management (analgesia) is necessary, as pain and discomfort in the wound area (sternum) were the main reasons for poor sleep quality following CS. Given that nurses maintain the most continuous interaction with patients after surgery, nurse‐led interventions for delirium prevention, especially those addressing modifiable sleep‐related factors may offer a more feasible and cost‐effective alternative to time‐ and resource‐intensive multidisciplinary protocols.

## 6 Conclusions

Poor sleep quality, insomnia, and the presence of sleep‐disordered breathing are the most common PSDs and are significant risk factors of POD after CS. Patients without POD tend to return to their preoperative sleep quality within about 1 month post‐surgery, whilst those with POD experience longer recovery, potentially taking 3–6 months to achieve a similar return to baseline sleep quality.

## Author Contributions

Conceptualization: H.V., P.D., L.B.R., F.M., M.R., and S.F.Q. Literature review search and extractions: H.V. and K.F. Study design and methodology: H.V. and F.M. Data curation and management: H.V. and K.F. Visualization: H.V. Software: H.V. and K.F. Statistical programming and validation: H.V. and K.F. Supervision: P.D., L.B.R., F.M., M.R., and S.F.Q. Formal Analysis: H.V. and K.F. Writing – original draft: H.V. Writing – review and editing: H.V., K.F., P.D., L.B.R., F.M., M.R., and S.F.Q.

## Conflicts of Interest

The authors declare no conflicts of interest.

## Supporting information


**Data S1:** Supplementary Information.

## Data Availability

The data that supports the findings of this study are available in the Supporting Information of this article.
