# Supplementary material for: Association Between Perioperative Sleep Disorders and Post‐Operative Delirium in Cardiac Surgeries: A Systematic Review and Meta‐Analysis
Source: J Sleep Res. 2025 Nov 8;35(3):e70241. doi: 10.1111/jsr.70241 (PMC13193506; doi:10.1111/jsr.70241)
Supplement: Supplementary file 1 — Data S1: Supplementary Information. [file JSR-35-e70241-s001.docx]

**Supplement files**

Contents

[Keywords used for searching databases. 2](#_Toc204290390)

[Keywords used for searching databases Updated July/2025 2](#_Toc204290391)

[Sleep dimension definitions 3](#_Toc204290392)

[Supplement tables: results of risk of bias assessment 7](#_Toc204290393)

[Results of sensitivity analysis (meta-analysis) 9](#_Toc204290394)

[Summary of included observational and interventional studies. 11](#_Toc204290395)

[PRISMA checklist 22](#_Toc204290396)

[References 24](#_Toc204290397)

# Keywords used for searching databases.

WOS: Sleep* OR "sleep disturbance" AND cardiac OR cardiovascular AND "post-operative delirium" OR "postoperative delirium" OR delirium

EMBASE: (sleep* OR 'sleep disturbance'/exp OR 'sleep disturbance') AND ('cardiac'/exp OR cardiac OR 'cardiovascular'/exp OR cardiovascular) AND ('post-operative delirium'/exp OR 'post-operative delirium' OR 'postoperative delirium'/exp OR 'postoperative delirium' OR 'delirium'/exp OR delirium)

PubMed: ((Sleep* OR "Sleep"[Mesh] OR "sleep disturbance" OR "Sleep Wake Disorders"[Mesh]) AND (cardiac OR "Cardiovascular Surgical Procedures"[Mesh] OR cardiovascular)) AND ("post-operative delirium" OR "postoperative delirium" OR delirium)

CINAHL: Sleep* OR "sleep disturbance" AND cardiac OR cardiovascular AND "post-operative delirium" OR "postoperative delirium" OR delirium

Scopus: ( sleep* OR "sleep disturbance" ) AND ("cardiac" OR cardiac OR "cardiovascular" OR cardiovascular ) AND ( "post-operative delirium" OR "postoperative delirium" OR delirium )

# Keywords used for searching databases Updated July/2025

CINAHL: ((MH "Delirium" OR "post-operative delirium" OR "postoperative delirium" OR delirium OR MH "Postoperative Period")) AND ((sleep* OR sleep disturbance OR sleep quality OR MH "Sleep Pattern Disturbance (NANDA)" OR MH "Sleep Disorders" OR MH "Insomnia") AND (cardiac OR cardiovascular OR MH "Cardiac Surgery" OR MH "Surgery, Cardiovascular")

PubMed: (("Delirium"[Mesh] OR "postoperative delirium" OR "post-operative delirium" OR delirium) AND ("Sleep Initiation and Maintenance Disorders"[Mesh] OR "Sleep"[Mesh] OR Sleep Disorders OR "Sleep Wake Disorders"[Mesh] OR sleep* OR "sleep disturbance" OR "sleep quality") AND ("Cardiovascular Surgical Procedures"[Mesh] OR "Cardiac Surgical Procedures"[Mesh] OR cardiac OR cardiovascular))

WOS: TS=(("postoperative delirium" OR "post-operative delirium" OR delirium) AND (sleep* OR "sleep disturbance" OR "sleep quality" OR "Sleep Wake Disorders" OR "Insomnia" OR "Sleep Pattern Disturbance ") AND (cardiac OR cardiovascular OR "cardiac surgery" OR "cardiovascular surgery" OR Cardiovascular Surgical Procedures))

EMBASE: (sleep* OR 'sleep disturbance'/exp OR 'sleep quality' OR 'sleep disorder'/exp OR 'sleep Wake Disorders'/exp) AND ('cardiac surgery'/exp OR 'cardiac'/exp OR cardiac OR 'cardiovascular'/exp OR cardiovascular OR 'Cardiovascular Surgical Procedures'/exp) AND ('postoperative delirium'/exp OR 'post-operative delirium' OR 'postoperative delirium' OR 'delirium'/exp OR delirium)

Scopus: ( "postoperative delirium" OR "post-operative delirium" OR delirium ) AND ( sleep* OR "sleep disturbance" OR "sleep quality" OR “sleep quality” OR “insomnia” OR "Sleep Wake Disorders" OR "Sleep Pattern Disturbance ") AND ( cardiac OR cardiovascular OR "cardiac surgery" OR "cardiovascular surgery" OR “Cardiovascular Surgical Procedures”)

# Sleep dimension definitions

Total sleep time (TST): The total duration spent in the state of sleep over a specific period, typically measured in hours and minutes excluding any periods of wakefulness (1).

Sleep onset latency (SL): Sleep latency, or sleep onset latency (SOL), refers to the duration of time it takes for an individual to transition from wakefulness to sleep after getting into bed. Optimally, the duration it takes to fall asleep, known as sleep latency, should range from 10 to 20 minutes (2).

Sleep efficiency (SE): Sleep efficiency (SE), often measured as the proportion of total sleep time to time spent in bed, is a key factor in the study and treatment of insomnia (3).

Wakefulness after sleep onset (WASO): This term refers to intervals of being awake that occur after a person has fallen asleep. This indicator quantifies the level of alertness, specifically omitting the period of alertness that occurs before falling asleep. WASO time provides a more accurate representation of sleep fragmentation (4).

Apnea-hypopnea index (AHI): The apnea-hypopnea index (AHI) represents the mean number of apneas and hypopneas that occur per hour of sleep. The American Academy of Sleep Medicine (AASM) classifies sleep apnea into three categories based on the number of occurrences per hour: normal (<5), mild (5-15 events/hour), moderate (15-30 events/hour), and severe (> 30 events/hour) (5) .

Specific descriptions of the measures are as follows:

Pittsburgh Sleep Quality Index (PSQI): A 19-item self-rated questionnaire about sleep quality (sleep efficiency, onset latency, duration, …) in the last month. A global score of more than 5 is suggested to be a clinical cutoff for low sleep quality and may indicate a need for clinical follow-up (6).

Epworth Sleepiness Scale (ESS): An 8-item self-rated scale that asks patients to rate (0 [no chance]–3 [high chance]) different potential daytime scenarios in which they may feel they want to doze off or fall asleep. A total score greater than or equal to 10 indicates excessive sleepiness during the daytime and may need to be managed clinically (7).

Insomnia Severity Index (ISI): A 7-item self-rated questionnaire that asks patients about their insomnia (in the last 2 weeks) and symptoms of insomnia. A global score of 8–14 indicates the threshold of insomnia, and a score greater than 14 indicates clinical insomnia, which requires clinical follow-up (8).

Richards-Campbell Sleep Questionnaire (RCSQ): A 5-item self-rated questionnaire (0-100 each item) that asks patients about their sleep quality last night (9). An average score of 50 or more could be considered as good sleep quality in an intensive care unit (10).

Polysomnography (PSG): A comprehensive objective sleep assessment (the gold standard) that measures neurophysiologic, cardiorespiratory, and other physiological variables simultaneously (11, 12). This technique could be performed in sleep clinics or patients’ homes (portable devices). The PSG measures electroencephalogram (via electrodes on head), electro-oculogram (for eye movements; via eye electrodes), and electromyogram (via electrodes on different part of body). These PSG measurements are necessary for determining sleep-wake stages. Additionally, PSG measures different dimensions of sleep e.g. total sleep time (TST), sleep onset latency (SL), sleep efficiency (SE), wakefulness after sleep onset (WASO), apnea-hypopnea index (AHI), and oxygen desaturation index (ODI). Supplement 3 includes the definitions.

American Academy of Sleep Medicine (AASM) (13) classified the sleep stages as non-rapid eye movement (NREM; which has 3 stages N1, N2, N3) and rapid eye movement (REM) (see supplement 3). Additionally, The AASM International Classification of Sleep Disorders (14) categorized sleep disorders into broad groups based on underlying causes, symptoms, and diagnostic criteria (Table 2).

| **Supplement Table – Sleep disorders according to American Academy of Sleep Medicine** | | | | | | |
| --- | --- | --- | --- | --- | --- | --- |
| **Category** | **Sleep Stages Affected** | **Sleep Latency** | **Sleep Duration** | **Sleep Efficiency** | **WASO (Wake After Sleep Onset)** | **AHI (Apnea-Hypopnea Index)** |
| Sleep-Related Breathing Disorders (e.g., Obstructive Sleep Apnea) | - REM sleep is often fragmented due to apneas, and it may be reduced.  - N3 (deep sleep) can also be reduced. | - Sleep latency may be normal or slightly prolonged. | - Total sleep time is often reduced. | - Efficiency is decreased due to frequent awakenings and apneas. | - WASO is increased due to repeated awakenings during the night. | - AHI is elevated (>5 per hour), indicating frequent apneas and hypopneas. |
| Insomnia (Chronic/Short-Term) | - Decreased time in N3 (deep sleep) and REM sleep.  - Increased time in N1 (light sleep). | - Increased sleep latency is a hallmark of insomnia. | - Reduced sleep duration, often <6 hours. | - Low sleep efficiency (<85%), as individuals spend more time awake in bed. | - WASO is increased, as insomnia leads to frequent nighttime awakenings. | - AHI is normal, as apneas/hypopneas are not typically present. |
| Central Disorders of Hypersomnolence (e.g., Narcolepsy, Idiopathic Hypersomnia) | - Narcolepsy: REM sleep onset occurs very quickly (sleep-onset REM periods, or SOREMPs).  - NREM sleep stages can be disrupted, especially in narcolepsy. | - Short sleep latency (in narcolepsy, <8 minutes).  - In idiopathic hypersomnia, latency may be normal but with excessive sleepiness. | - Increased daytime sleepiness despite often normal or increased total sleep time. | - Efficiency may be normal but is often impaired by fragmented sleep in narcolepsy. | - WASO can be increased due to fragmented sleep in narcolepsy, but is often normal in idiopathic hypersomnia. | - AHI is normal, as these are not breathing disorders. |
| Circadian Rhythm Sleep-Wake Disorders (e.g., Shift Work Disorder, Delayed Sleep Phase Syndrome) | - REM and NREM sleep are typically normal but can be reduced due to misaligned sleep schedules.  - Circadian misalignment may result in fragmented REM sleep. | - Delayed sleep latency if sleeping at an inappropriate circadian time. | - Total sleep duration may be reduced due to misalignment with circadian rhythms or work schedules. | - Reduced sleep efficiency due to difficulty falling asleep and frequent awakenings. | - WASO may be increased due to frequent nighttime awakenings caused by circadian misalignment. | - AHI is normal, unless there is a comorbid sleep-related breathing disorder. |
| Parasomnias (e.g., Sleepwalking, REM Sleep Behavior Disorder) | - Sleepwalking occurs during NREM (N3) sleep.  - REM Sleep Behavior Disorder (RBD) involves a lack of muscle atonia during REM sleep, leading to vivid dream enactment. | - Sleep latency is typically normal in parasomnias. | - Total sleep duration may be normal, though disrupted by parasomnia events. | - Efficiency is often normal, but sleep fragmentation may occur in REM-related parasomnias. | - WASO is increased if parasomnia episodes lead to full awakening or disruption of sleep. | - AHI is normal, unless there is a comorbid breathing disorder. |
| Sleep-Related Movement Disorders (e.g., Restless Legs Syndrome, Periodic Limb Movement Disorder) | - NREM sleep (especially N2 and N3) is often disrupted by movements.  - REM sleep may also be fragmented. | - Sleep latency is often increased due to discomfort in Restless Legs Syndrome. | - Total sleep duration may be reduced due to repeated arousals or discomfort. | - Reduced sleep efficiency due to frequent movement-induced awakenings. | - WASO is increased due to frequent nighttime awakenings caused by limb movements. | - AHI is normal, unless there is a comorbid breathing disorder. |

**Delirium assessment tools**

| **Tool Name** | **No. of Items** | **Validated** | **Reliability** |
| --- | --- | --- | --- |
| CAM (Confusion Assessment Method) (15) | 4 | Yes | Yes |
| CAM-ICU (CAM for ICU) (16) | 4 | Yes | Yes |
| 3D-CAM (3-Min Diagnostic CAM) (17) | 22 | Yes | Yes |
| DRS (Delirium Rating Scale) (18) | 10 | Yes | Yes |
| DRS-R-98 (Revised DRS) (19) | 16 | Yes | Yes |
| DSM-IV Criteria (20) | N/A (generally 4 criteria) | Yes | Yes |
| Clinician Diagnosis | N/A | Not standardized | No |
| NEECHAM Confusion Scale (21) | 9 | Yes | Yes |
| DOS (Delirium Observation Screening Scale) (22) | 25 (also another scale with 13-item) | Yes | Yes |

# Supplement tables: results of risk of bias assessment

**Final risk of bias (ROB) score; > 85% Low; 75 – 85% Moderate; <75% High**

Cohort studies

| Study | Q1 | Q2 | Q3 | Q4 | Q5 | Q6 | Q7 | Q8 | Q9 | Q10 | Q11 | Final score | ROB |
| --- | --- | --- | --- | --- | --- | --- | --- | --- | --- | --- | --- | --- | --- |
| Ibala et al | Yes | Yes | Yes | Yes | No | Yes | Yes | Yes | Yes | Yes | Yes | 91% | Low |
| Oldham et al | Yes | Yes | Yes | Yes | No | Yes | Yes | Yes | Yes | N/a | Yes | 82% | Moderate |
| Wang et al | Yes | Yes | Yes | Yes | Yes | Yes | Yes | Yes | Yes | Yes | Yes | 100% | Low |
| Roggenbach et al | No | Yes | Yes | Yes | Yes | Yes | Yes | Yes | Yes | N/a | Yes | 82% | Moderate |
| Zhang et al | No | Yes | No | Yes | N/a | Yes | Yes | Yes | Yes | N/a | Yes | 64% | High |
| Cheraghi et al | No | Yes | Yes | No | No | Yes | Yes | UC | Yes | N/a | Yes | 55% | High |
| Varga-Martínez et al | Yes | Yes | Yes | Yes | No | Yes | Yes | Yes | Yes | N/a | Yes | 82% | Moderate |
| Atalan et al | Yes | Yes | Yes | Yes | No | Yes | Yes | Yes | Yes | N/a | Yes | 82% | Moderate |
| Chen et al | Yes | Yes | Yes | Yes | No | Yes | Yes | Yes | Yes | N/a | Yes | 82% | Moderate |
| Lin et al | Yes | Yes | Yes | Yes | Yes | Yes | Yes | Yes | Yes | N/a | Yes | 91% | Low |
| Javaherforooshzadeh et al | Yes | Yes | Yes | Yes | Yes | UC | N/a | Yes | Yes | Yes | Yes | 82% | Moderate |
| Tafelmeier et al | Yes | Yes | Yes | Yes | Yes | UC | Yes | Yes | Yes | Yes | Yes | 91% | Low |
| Rivas et al | Yes | Yes | Yes | Yes | Yes | UC | Yes | Yes | Yes | Yes | Yes | 91% | Low |
| Koster et al | Yes | Yes | No | No | No | UC | Yes | UC | Yes | Yes | Yes | 55% | High |
| Huang et al | UC | Yes | Yes | Yes | Yes | Yes | Yes | Yes | UC | UC | Yes | 73% | High |
| Varga-Martinez et al | UC | Yes | No | Yes | Yes | UC | Yes | UC | Yes | Yes | Yes | 64% | High |
| Cai et al | Yes | Yes | Yes | Yes | Yes | UC | Yes | Yes | Yes | Yes | Yes | 91% | Low |
| Chen et al | Yes | Yes | UC | Yes | Yes | UC | Yes | Yes | Yes | Yes | Yes | 82% | Moderate |
| Kim et al | Yes | Yes | No | Yes | No | Yes | Yes | Yes | Yes | Yes | Yes | 82% | Moderate |

**Final risk of bias (ROB) score; > 85% Low; 75 – 85% Moderate; <75% High**

Cross sectional studies

| Study | Q1 | Q2 | Q3 | Q4 | Q5 | Q6 | Q7 | Q8 | Final score | ROB |
| --- | --- | --- | --- | --- | --- | --- | --- | --- | --- | --- |
| Simeone et al | Yes | Yes | No | Yes | Yes | Yes | Yes | Yes | 87.5% | Low |
| Mahmoudi et al | Yes | Yes | Yes | Yes | Yes | No | Yes | No | 87.5% | Low |

**Final risk of bias (ROB) score; > 85% Low; 75 – 85% Moderate; <75% High**

Randomized controlled trials

| Study | Q1 | Q2 | Q3 | Q4 | Q5 | Q6 | Q7 | Q8 | Q9 | Q10 | Q11 | Q12 | Q13 | Final score | ROB |
| --- | --- | --- | --- | --- | --- | --- | --- | --- | --- | --- | --- | --- | --- | --- | --- |
| Fazlollah et al | Yes | Yes | Yes | No | No | UC | Yes | Yes | Yes | Yes | Yes | Yes | Yes | 77% | Moderate |
| Shorofi et al | Yes | Yes | Yes | No | Yes | UC | Yes | Yes | Yes | Yes | Yes | Yes | Yes | 85% | Moderate |
| Dianatkhah et al | Yes | Yes | Yes | Yes | UC | Yes | Yes | No | Yes | Yes | No | Yes | Yes | 77% | Moderate |
| Qu et al | Yes | Yes | Yes | Yes | Yes | UC | Yes | No | Yes | Yes | Yes | Yes | Yes | 85% | Moderate |
| Huang et al (18) | Yes | Yes | Yes | Yes | Yes | Yes | Yes | UC | Yes | Yes | Yes | Yes | Yes | 92% | Low |
| Huet et al | Yes | Yes | UC | Yes | Yes | Yes | Yes | Yes | Yes | Yes | Yes | Yes | Yes | 92% | Low |
| Turan et al | Yes | Yes | Yes | Yes | Yes | Yes | Yes | Yes | No | Yes | Yes | Yes | Yes | 92% | Low |
| Freedman et al | Yes | Yes | Yes | Yes | Yes | Yes | Yes | No | Yes | Yes | Yes | Yes | Yes | 92% | Low |
| Yang et al | Yes | Yes | Yes | Yes | Yes | Yes | Yes | No | Yes | Yes | Yes | Yes | Yes | 92% | Low |
| Fang et al | Yes | Yes | Yes | Yes | Yes | UC | Yes | Yes | Yes | Yes | Yes | Yes | Yes | 92% | Low |

*UC: unclear

**Final risk of bias (ROB) score; > 85% Low; 75 – 85% Moderate; <75% High**

Quasi experimental studies

| Study | Q1 | Q2 | Q3 | Q4 | Q5 | Q6 | Q7 | Q8 | Q9 | Overall | ROB |
| --- | --- | --- | --- | --- | --- | --- | --- | --- | --- | --- | --- |
| Zhang et al | Yes | Yes | Yes | UC | Yes | Yes | Yes | Yes | Yes | 89% | Low |
| Lin et al | Yes | Yes | No | Yes | Yes | Yes | Yes | Yes | Yes | 89% | Low |

# Results of sensitivity analysis (meta-analysis)


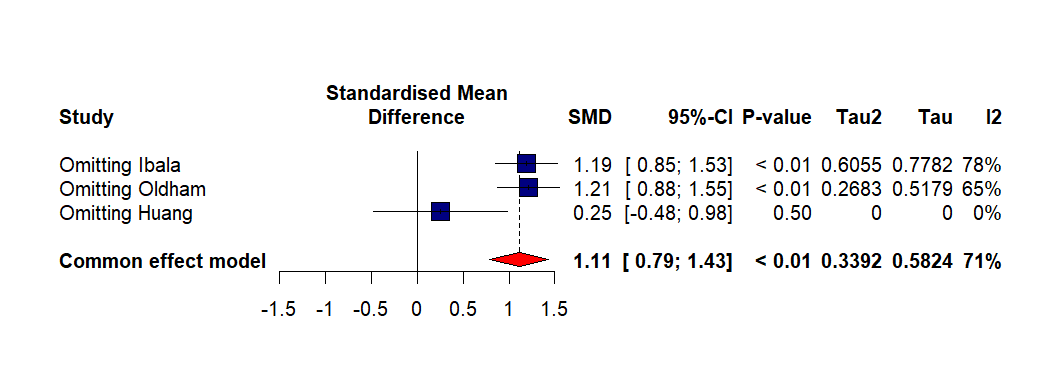


Supplement Figure 1 – sensitivity analysis on the Association of Pittsburg Sleep Quality Index (PSQI) with Post-operative Delirium (POD) in Cardiac Surgery Patients


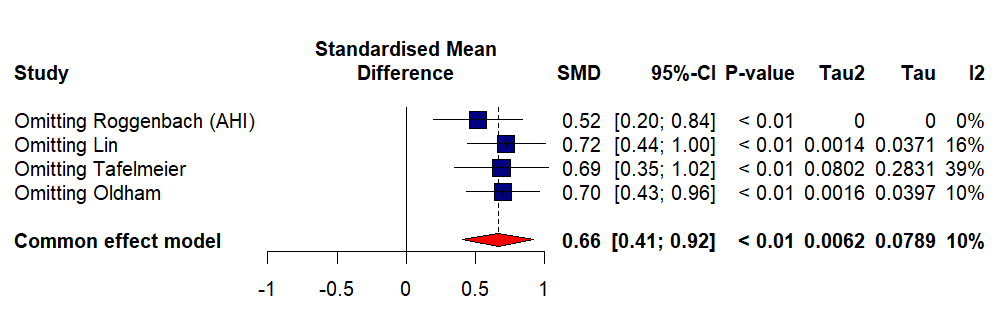


Supplement Figure 2 – sensitivity analysis on the Association of Apnea-hypopnea index (AHI) with POD in Cardiac Surgery Patients.


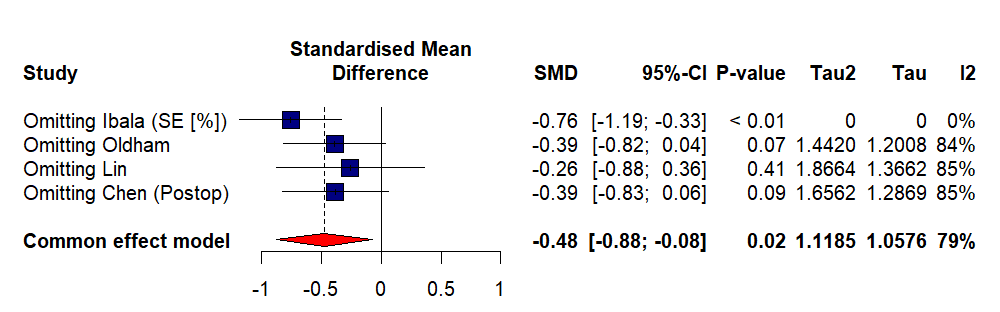


Supplement Figure 3 – sensitivity analysis on the Association of Sleep Efficiency (SE [%]; measured by PSG) with POD in Cardiac Surgery Patients.


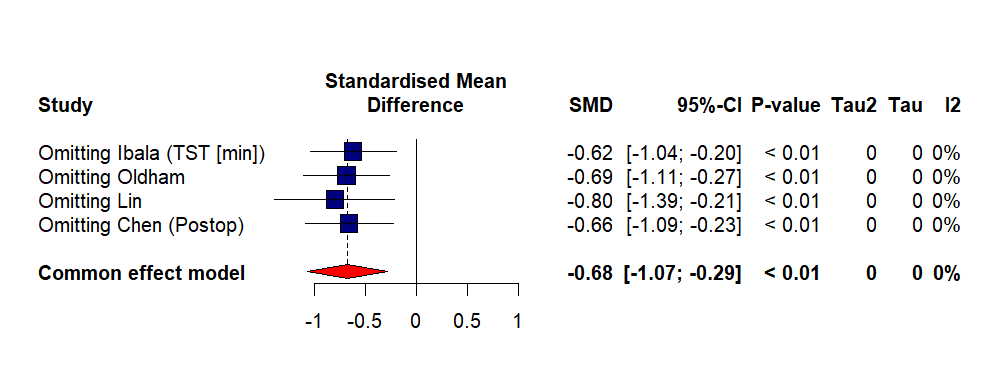


Supplement Figure 4 – sensitivity analysis on the Association of Total Sleep Time (TST [min]; measured by PSG) with POD in Cardiac Surgery Patients.


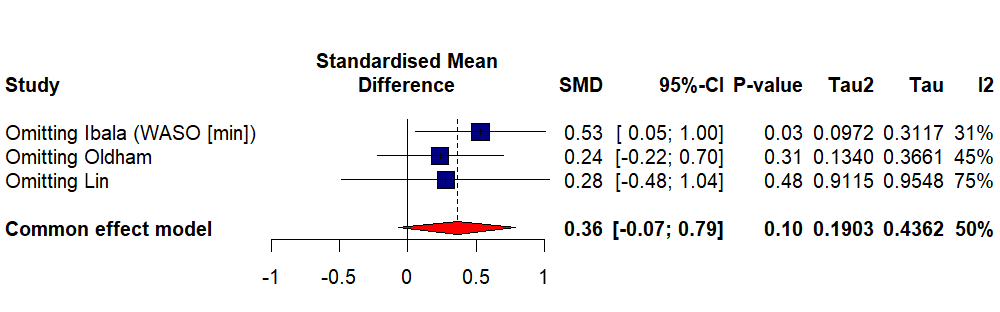


Supplement Figure 5 – sensitivity analysis on the Association of Wakefulness after Sleep Onset (WASO [min]; measured by PSG) with POD in Cardiac Surgery Patients

# Summary of included observational and interventional studies.

| Table 1 - summary of included observational studies | | | | |
| --- | --- | --- | --- | --- |
| Author (year), Country | Study design & population | Sleep measurement/s and outcome  (delirious compared to non-delirious) | Delirium assessment and outcome* | Major key findings |
| Ibala (2021)  USA (23) | Cohort.  Patients≥ 60 years  Elective CPB | Polysomnography (1-day **preop**)  PSQI (1-day **preop**).  Sleep duration: longer ^*^  SE: higher^*^  Onset latency: lower^*^  Stage N1 latency: lower^*^  Stage N2 latency: lower^*^  Stage N2 duration: higher^*^  PSQI: higher^≠^ | CAM  Twice daily  For 3 days | Longer duration of sleep REM, and decreased sleep onset latency are associated with POD.  No significant difference observed in terms of preoperative cognitive function, anxiety and mental health, anesthesia, and surgery duration and techniques. |
| Wang (2020)  China (25) | Prospective, cohort.  Patients ≥18 years  On-pump valve surgery | PSQI (1-month **preop**).  Sleep disturbance in univariate and multivariate (OR=3.22) ^*^ | CAM-ICU  First assessment 24 hours after surgery, then twice daily  For 7 days | Age was another predictor of delirium in multivariate regression (OR= 1.10). In univariate analysis, intraoperative infusion volume (843 cc), postop intubation time (1384 min), and time of ICU stay (3.6 days) were predictors of delirium.  No significant differences in baseline cognitive function were noted. |
| Roggenbach (2014)  Germany (26) | Prospective, cohort.  Patients ≥18 years,  CABG or valve on-or-off pump surgery  without diagnosed SA and with no cognitive impairment. | Polygraph for sleep-breathing pattern (1-day **preop**).  AHI: higher^*^  Oxygen desaturation: lower^*^  Multivariate analysis: AHI was an independent risk factor (OR=1.05) ^*^ | CAM-ICU  Twice daily.  Assessment duration: NA | Older ages (OR=1.16), smoking (OR=32.37), and intraoperative blood transfusion (OR=1.55) were significant predictors of delirium. |
| Oldham (2021)  USA (27) | Cohort.  Patients aged 55-89 years  Candidates for surgical aortic valve replacement. | PSQI, ISI, ESS, STOP-Bang, and polysomnography (2–3 weeks **preop**).  ISI: higher^≠^  PSQI: higher^≠^  ESS: higher^≠^  STOP-bang: lower^≠^  Total sleep time: lower^≠^  REM time: lower^≠^  N1 time: higher^≠^  N2 time: lower^*^  N3 time: higher^≠^  SE time: lower^≠^  AHI time: higher^≠^  SL time: higher^≠^  WASO time: higher^≠^ | 3D-CAM and DRS  Assessment Frequency: N/A  Assessment duration: N/A | Age was significantly correlated with SE. No significant correlation between variables and AHI or SE was noted. |
| Zhang (2015)  China (28) | Prospective cohort.  Patients ≥18 years  Candidates for CABG who were admitted to ICU after surgery. | **Postop** Patient’s self-reported sleep quality.  Sleep quality: lower^*^  In multivariate analysis, poor sleep quality (OR=5.001) significantly predicted delirium. | CAM-ICU.  Surgery day to day6 postop.  Assessment Frequency: N/A | Preoperative AF (OR= 3.957) and cognitive impairment (measured by MMSE; OR= 3.231), and postoperative electrolyte disturbance (OR= 2.095) are other predictors of delirium.  Patients with delirium had significantly higher hospital and ICU length of stay but no significant differences in mortality were noted. |
| Cheraghi (2016)  Iran (29) | Prospective cohort.  Patients ≥18 years  candidates for selective open-heart surgery  with no alcohol or drug disorders | PSQI (second day **postop**).  42.1% of patients who had poor sleep quality developed delirium ^*^ | CAM-ICU  Twice daily  Day 2-5 after surgery. | Older ages (>60) and male sex were more likely to develop delirium. |
| Varga-Martínez (2023)  Spain (31) | Prospective observational, cohort.  Patients ≥18 years  who underwent cardiac surgery. | Self-reported sleep disturbance (falling asleep and staying asleep) and having nightmares,  **3 years** after hospital discharge(**postop**).  Sleep disturbances and nightmares: higher^*^  Problems with falling and staying asleep: higher^*^ | CAM-ICU  Daily during hospital stay.  Assessment in each shift  Overall: 25.6% (55/215). | Patients who were diagnosed with delirium during hospital stays had significantly higher hospital readmission rates, memory problems, concentration problems, confusion, emotional problems, difficulty returning to the job, and mobility restrictions. |
| Atalan (2013)  Turkey (32) | Prospective, longitudinal, cohort.  Candidates for cardiac surgery using CPB. | PSQI (12-months **postop**).  PSQI: higher^*^ at 12-month follow-up (OR=10.85). | DSM-IV and CAM-ICU  Daily  Assessment duration: N/A | Patients who experience delirium have significantly lower MMSE (cognitive dysfunction) before hospital discharge and after 6 and 12 months of follow-up. Delirious patients had significantly higher ICU (mean = 3 days) and hospital stays (mean = 10 days). |
| Chen (2020)  China (33) | Prospective, cohort.  Patients aged ≥18 years  Cardiac surgery using CPB. | Polysomnography (1-day **postop**).  Total sleep time: lower^≠^  Sleep efficacy: lower^≠^  N1 stage %: higher^≠^  N2 stage %: lower^≠^  N3 stage %: lower^≠^  REM stage %: lower^*^  Absence of REM sleep: higher^*^  Absence of N3 sleep: higher^≠^  Atypical sleep: higher^*^ | CAM-ICU  Three times a day.  Until postoperative day 5 | There was no significant difference in the duration of surgery, the time of CPB, or the duration of the ICU stay between the two groups. |
| Lin (2023)  China (34) | Prospective, cohort.  Patients aged ≥18  Candidates of valve surgeries without cognitive dysfunction (MMSE<24). | Polysomnography (1-day **preop**)  RCSQ (day of surgery **preop**)  Presence of sleep apnea based on International Classification of Sleep Disorders.  RCSQ: lower^≠^  SE: lower^*^  TST: lower^*^  Slow wave sleep: lower^*^  NREM arousal index: higher^*^  REM arousal index: higher^≠^  TST arousal index: higher^*^  N1 stage: higher^≠^  N2 stage: higher^≠^  OSA: higher^*^  Lowest oxygen saturation: lower^*^  Sleep apnea: higher^≠^  AHI: higher^≠^  In multivariate regression analysis, after adjusted for age, sex, and BMI, duration of surgery and length of mechanical ventilation, percent of Stage N1 sleep (OR= 1.103), slow wave sleep (OR= 0.647), percent of REM sleep (OR= 0.872), NREM-arousal index (OR= 1.093), and TST-arousal index (OR= 1.114) significantly predict delirium occurrence. | CAM-ICU  Three times a day  Assessment until extubation | Older ages (>57), longer duration of surgery (>284mon), longer duration of mechanical ventilation (>19h), and longer ICU stay (>45h) were significant risk factors of delirium. No significant difference in HRV variables between groups was noted. |
| Javaherforooshzadeh (2022)  Iran (80) | Prospective, cohort.  306 patients aged ≥18  Underwent elective major cardiac surgery | STOP-BANG (night of surgery **preop)**  POD incidence: ^≠^  Low risk 2/33 (6.1%)  Intermediate risk 2/100 (2.0%)  High risk 4/173 (2.3) | Delirium diagnosis tool: NA  Assessment frequency: NA  Assessment duration: about 10 days | Study showed patients with high risk of OSA have significantly more hypertension incidence postop but lower ICU readmission and wound infection. |
| Tafelmeier (2019)  Germany (82) | Prospective, cohort.  141 patients aged 18-85 years  Underwent elective CABG with or without valve surgery | Pulse oximetry, nasal flow and thoracic breathing effort (night before surgery **preop**)  AHI= higher^*^  Obstructive apnea index= higher^≠^  OSA n%= lower^≠^  Central apnea index= higher^*^  CSA n%= higher^*^  Sleep disordered breathing n%= higher^*^  Multivariate:  CSA: OR=4.33 (1.17-16.11) ^*^ | CAM-ICU  Assessment frequency: NA  For 3 days | In multivariate regression age⩾70 years, preoperative need for loop diuretics, CSA, history of transient ischemic attack or stroke and heart failure were risk factors for POD. |
| Koster (2009)  Netherlands (84) | Prospective, cohort.  112 patients≥ 45 years  Underwent cardiac surgery  9 patients lose to follow-up | Self-reported sleep disturbance (1 year **postop**)  Sleep disturbances: higher^*^ | DSM-IV  Assessment frequency: NA  Assessment duration: NA | Patients with POD showed higher sleep disturbance, concentration problem, memory problem, dependency in mobility and emotional problems 1 year after the surgery but the difference in sleep disturbance was significant. |
| Huang (2024)  China (85) | Retrospective cohort  194 patients ≥ 60 years  Underwent cardiac surgery | PSQI (monthly for 2 years **preop**)  Sleep disorder: higher (OR= 3.772) ^*^  PSQI: higher (OR= 1.207) ^*^  Number of months of high PSQI: higher (OR= 1.102) ^*^ | CAM, CAM-ICU  Daily  For 4 days | Study showed long-term low sleep and a decline in sleep quality is associated with higher POD incidence. |
| Varga-Martínez (2021)  Spain (86) | Prospective, cohort and model development  689 patients (validation group= 344, development group= 245) >18 years  Underwent cardiac surgery with cardiopulmonary bypass | History of insomnia needing medical treatment (**preop**)  Insomnia:  Development group: higher^*^  Validation group: higher^*^ | CAM-ICU  Assessment done every shift  For the duration of ICU stay | The model for predicting POD included four preoperative risk factors including age> 65, MMSE score, insomnia needing medical treatment and low physical activity. |
| Simeone (2018)  Italy (87) | Cross-sectional  89 patients ≥ 18 years  Underwent heart surgery | Self-reported sleep disorder (**timing not reported**)  Sleep disorders: higher^*^ (relative risk ratio: 5.493 95CI%= 1.255-24.047) | CAM-ICU  Assessment frequency: NA  Assessment duration: NA | In logistic regression this study found that age, duration of mechanically assisted ventilation, location regarding sunlight and sleep disorders were related to POD. |
| Cai (2025) (74) | Prospective cohort; 216 patients with acute aortic dissection surgery. Divided into good sleep (PSQI ≤7, n=103) vs poor sleep (PSQI >7, n=113) groups based on pre-op sleep survey. Monitored postoperative outcomes. | Pittsburgh Sleep Quality Index on admission. Median PSQI was 5 in good-sleep vs 9 in poor-sleep group. Delirium vs non: POD occurred more frequently in poor sleepers (exact rates not given, but significant p<0.05). Poor-sleep group also had longer mechanical ventilation (62 more hours on average) and ICU stays. | Delirium: Assessed by CAM/CAM-ICU during hospitalization. POD incidence was significantly higher in the poor sleep group (no POD in 90% of good sleepers vs 78% of poor sleepers, approximate from text). In multivariate analysis, poor pre-op sleep quality (OR ~11.6) and POD (OR ~5.1) each independently predicted prolonged ventilation. | Preoperative poor sleep quality was associated with a higher risk of POD, prolonged mechanical ventilation, and longer ICU stay. Patients with poor sleep had ~3.5× higher odds of in-hospital death and ~5× higher odds of POD than good sleepers. Authors suggest simple sleep quality screening can identify high-risk patients and improve prognostic stratification. |
| Chen (2024) (75) | Retrospective cohort (MIMIC-IV ICU database); 4,286 cardiac surgery patients ≥65 y. Investigated relationship between nutritional status (GNRI) and POD risk, adjusting for comorbidities. | Sleep: Medical history/record of OSA. Delirious vs non: Similar prevalence of pre-existing sleep apnea in both groups (~13% vs 11%, p=0.206). Sleep disruption is discussed as a modifiable risk (cites literature) but not measured in this study. | Delirium: Identified via chart review/ICU documentation (likely CAM-ICU or ICD codes). POD occurred in 659 patients (16.1%). Delirious patients had significantly lower GNRI (median 111.1 vs 113.4) and higher rates of malnutrition. | Malnutrition (GNRI ≤98) was associated with higher POD odds (OR 1.83). In multivariable models, GNRI remained an independent negative predictor for POD. Authors note that many delirium risk factors are modifiable (e.g. “sleep promotion, family support, environmental interventions”) and suggest nutritional optimization plus standard delirium prevention (including sleep improvement) to reduce POD. |
| Kim (2024) (76) | Prospective cohort study; older adults (>65) undergoing major elective surgery (n=195) | Medical history/record of OSA | Nu-DESC.  POD occurred in 34.4%. | Preoperative sleep disorder was an independent risk factor for POD after cardiac surgery. |
| Mahmoudi (2025) (79) | Multiphase multicenter study; derived and tested a risk prediction tool (SDACS) for POD in 920 cardiac surgery patients. Phase 1: identified 136 risk factors via literature; Phase 2: Delphi consensus to prioritize predictors; Phase 3: prospective observation of 920 patients to validate predictors. | Sleep: Recorded objective sleep duration on post-op nights. Delirious vs Non: Among patients who slept ≤4.5 h on the first night, a very large proportion developed delirium (approximately 78% delirious vs 22% non-delirious). By contrast, those who slept >4.5 h rarely became delirious. “Poor sleep quality” (≤4–4.5 h sleep) on Night 1 had OR 9.08 for POD. (Poor second-night sleep was also significant, OR not given). No subjective sleep scales were used. | Delirium: Assessed after surgery using CAM-ICU twice daily (from POD1 onward). Incidence 53% (488/920). The final SDACS tool included 4 predictors: first-night poor sleep, chronic opioid use, benzodiazepine history, and hearing impairment. It showed excellent discrimination (AUC ~0.90). | Early postoperative sleep loss emerged as a powerful predictor of delirium in cardiac surgery patients. Patients getting <4–5 hours of sleep on the first ICU night had dramatically higher delirium rates. Along with avoiding certain meds (opioids, benzos) and addressing sensory deficits, preserving sleep is highlighted for delirium prevention. The new SDACS screening tool can help nurses identify high-risk patients early, prompting interventions (e.g. strategies to improve ICU sleep) to potentially reduce POD incidence. |

^*^ Significant, ^≠^ not significant, CSA = central sleep apnea

| Table 2 – summary of included interventional studies | | | | |
| --- | --- | --- | --- | --- |
| Author (year), Country | Study design & population | Sleep measurement/s and outcome | Delirium assessment and outcome** | Major key findings |
| Qu (2023)  USA (24) | RCT  (Dexmedetomidine[n=188] vs. placebo[n=206]).  Patients ≥ 60 years  Candidates for cardiac surgery  Planned to stay in the ICU for at least 24 hours. | PROMIS Sleep Disturbance short form (once; **preop** and **postop** 30, 90, 180 days)  History of sleep apnea.  Preoperative sleep disturbance score: ^≠^  History of sleep apnea: ^≠^  Dexmedetomidine did not show a significant difference in postoperative sleep disturbance score compared to placebo at 30, 60, and 180.  The sleep disturbance scores preoperatively were close to the postoperative scores at day 30 (50.5 in both groups). In both groups, the sleep disturbance score at day 60 and 180 was reduced by 2 (48.4) and 4 units (46.2), respectively. | CAM  Twice daily  3 days or until the hospital discharge whichever sooner | Dexmedetomidine can significantly prevent POD following cardiac surgeries but may have no impact on length of hospital stay, mortality, mid-term sleep disturbance, physical, and mental function. |
| Dianatkhah (2015)  Iran (30) | RCT  (Melatonin [3 mg, n=66] vs Oxazepam [10 mg, n=71], starting 3 days before surgery until hospital discharge).  Candidates for (on-pump) CABG without a history of sleep disorder or history of nervous system depressant | GSQS.  (6 and 5 days **preop** and 3 and 5 days **postop**)  Patients in Melatonin group had significantly better sleep quality after surgery compared to Oxazepam group.  No significant difference in delirium incidence was noted. | Clinical observation of trained nurses.  Assessment Frequency: N/A  Assessment duration: N/A | Melatonin, compared to oxazepam, results in a lower anxiety score and better sleep quality.  Pump and cross-clamp time were significantly higher in Melatonin group. Patients in the Melatonin group had significantly fewer hours of ICU stay. |
| Shorofi (2023)  Iran (35) | RCT  (from the 2^nd^ day postop: earplugs and eye mask [n=57] vs. routine care [n=57]).  Patients aged ≥18 years  Underwent CABG. | VSHSS (Daily up to 4-days **postop**)  Sleep disturbance score, supplementation, and effectiveness scores were significantly lower through day 1 to day 4 in intervention group (indicates better sleep quality). | NEECHAM  Daily  From day 2 to 5 postop. | Applying earplugs and an eye mask after CABG not only improves sleep quality but also reduces the incidence of delirium. No other significant risk factors were noted. |
| Fazlollah (2021)  Iran (36) | RCT  (foot reflexology massage [n=30] vs. control [n=30]).  Patients aged ≥18 years who underwent CABG. | RCSQ (**postop** day 1 and 2).  Foot massage intervention did not significantly improve sleep quality, latency, number of awakenings, or ease of return to sleep, compared to the control group. | DOS  Assessment Frequency: N/A  For 2 days | Foot massage interventions significantly reduced the pain intensity on days 1 and 2 after surgery, compared to the control group. |
| Huang (2024)  China (78) | RCT  (Intranasal insulin twice daily two days preop [n=36] vs. saline [n=35])  Patients aged 18-65 years  Underwent valve surgery with cardiopulmonary bypass | PSQI, deep sleep ratio, REM sleep ratio, number of awakenings, deep sleep continuity score, total sleep quality score (**preop,** before first intervention[T0], one day before surgery [T1], before anesthesia [T2])  Intervention vs. to control:  PSQI: T0^≠^, T1 lower^*^, T2 lower^*^  Deep sleep%: T1higher^*^, T2 higher^*^  Light sleep%: T1 lower^*^, T2 lower^*^  REM sleep%: T1 higher^*^, T2 higher^*^  Number of awakenings: T1 lower^*^, T2 lower^*^  Deep sleep continuity score: T1 higher^*^, T2 higher^*^  Total sleep quality score: T1 higher^*^, T2 higher^*^ | CAM-ICU  Twice daily  For 3 days | Patients receiving 20 U insulin twice daily from 2 days preop compared to saline, have reduced POD incidence and improved sleep quality. |
| Huet (2024)  France (79) | RCT  (Postop infusion of dexmedetomidine [N=165] vs. placebo [N=166])  Patients≥65 years  Underwent cardiac surgery with or without cardiopulmonary bypass | Sleep quality numerical scale, LSEQ (**postop up to 7 days**)  Intervention vs. control:  Intention to treat analysis:  Sleep quality: lower^≠^  LSEQ: lower^≠^  As treated analysis:  Sleep quality: higher^*^  LSEQ: lower^*^ | CAM-ICU  Twice daily  For 7 days | Although intervention with dexmedetomidine did not decrease POD, but overall sleep quality was higher compared to control group. |
| Turan (2020)  USA (81) | RCT (sub analysis)  (Dexmedetomidine [n=398] vs. placebo[n=396])  Patients aged 18-85 years who were candidates for CS with cardiopulmonary bypass. | Sleep Interference scale (90 days **postop**)  Not reported! | CAM-ICU  Twice daily  For 5 days | Dexmedetomidine did not significantly reduced the incidence of POD following CS. Also, dexmedetomidine caused longer ICU stay.  No association noted about OSA, atrial fibrillation and POD. |
| Zhang (2017)  China (71) | Prospective, before-after study  (protocol for nursing intervention)  278 patients ≥ 18 years  Underwent CABG | Monitoring sleep difficulties and providing related intervention (**postop**)  Quality of sleep: higher (significancy not specified) | CAM-ICU, DSR-R-98  Three times daily  For 7 days | Interventional nursing protocol reduced delirium incidence and severity as well as improved quality of sleep.  Sleep quality of and electric disturbances were independent postop risk factors. |
| Freedman (2025) (72) | Secondary analysis of an RCT dataset (MINDDS trial) in 394 cardiac surgery patients ≥60 y. Examined relationship between intraoperative EEG patterns and postoperative outcomes. (All patients were originally in a trial of night-time DEX vs placebo, but this analysis ignored group assignment and focused on EEG exposure.) | PROMIS. Delirious vs Non: Intra-op EEG alpha power was lower in those who developed delirium (mean ~10 dB vs ~11 dB; 0.8 dB lower, unadjusted p<0.01) – indicating deeper anesthetic EEG or more burst suppression in delirious patients. No patient-reported sleep data were analyzed. | Delirium: Assessed by CAM on postoperative days (delirium incidence 11.1%). Unadjusted, each +1 dB of alpha power during maintenance anesthesia was associated with 14% lower odds of POD. After adjusting for pre-op cognitive/physical health, EEG alpha was not an independent predictor. Secondary outcomes: alpha power did predict non-home discharge (adjusted) and fewer 30-day readmissions. | Patients with higher intraoperative EEG alpha power (a marker of lighter anesthesia or preserved brain activity) tended to have lower delirium rates, although this was confounded by patient health status. This suggests that overly deep anesthesia (suppressed EEG) might relate to POD. The study highlights that anesthesia-induced EEG changes are linked to delirium and other outcomes, but optimizing anesthetic depth alone may not prevent delirium once patient frailty is considered. |
| Yang (2025) (73) | Randomized controlled trial; middle-aged patients (45-65) undergoing elective on-pump cardiac surgery (n=76) | Actigraphy (TST, SE, WASO, No. of awakenings); INI group showed improved TST and SE on POD1 and POD3 | CAM-ICU, CAM, 4-AT; INI group had significantly lower POD incidence (17.1% vs. 38.9%, p=0.042) | Intranasal insulin reduced POD and improved sleep quality (TST, SE) and MMSE scores postoperatively |
| Lin (2025) (77) | Comparative intervention study; 159 elderly cardiac surgery patients (≥65 y). Control group (n=82) got routine peri-op care; Observation group (n=77) received a multifaceted POD prevention program (education, sleep protocols, early rehab, pain & nutrition management). | Sleep: Sleep quality was evaluated (method not fully described). After intervention, the observation group had significantly better sleep scores than controls (higher “sleep quality” score, F=219.2, p<0.001). Delirious vs Non: In controls, poor sleepers were more likely to become delirious (implied but data not shown). In the intervention group, sleep scores improved and delirium was less frequent, suggesting a link. | Delirium: Monitored daily (likely 3-D CAM/ CAM-ICU). POD incidence was 47.6% in control vs 24.7% with intervention (χ² = 8.976, p=0.003). The intervention also shortened hospital stay (12.6±3.0 vs 17.3±7.1 days) and reduced pain scores and ICU costs. | An evidence-based bundle of interventions significantly reduced delirium rates and improved outcomes in elderly cardiac surgery patients. Key elements included structured sleep enhancement (eg. noise reduction, sleep–wake scheduling) and aggressive pain/nutrition management. Improved sleep quality in the intervention group correlated with their lower delirium incidence. This underscores that optimizing postoperative care (especially sleep and pain management) can mitigate POD. |
| Fang (2024) (78) | Prospective RCT; 100 cardiac surgery patients (≥60 y) with pre-op sleep disorders (PSQI ≥8). Randomized to preoperative intranasal dexmedetomidine (DEX) vs placebo the night before surgery. All received standard care otherwise. | Sleep: Baseline PSQI median ~9 (all had poor sleep by criteria). Outcome: Night of intervention, DEX group saw a sharp drop in insomnia incidence (from 36% of patients to 10%, p=0.002) vs. placebo (32%→26%, p=0.51). This suggests DEX improved subjective sleep quality markedly. No objective sleep measurement was reported, but DEX patients were monitored in a special sleep room overnight. | Delirium: Assessed with CAM-ICU in ICU and CAM on wards. POD incidence was 12% with DEX vs 30% with placebo (p=0.027). (Absolute risk 18% lower with DEX.) Delirium typically occurred on POD1; DEX group had shorter episodes when delirium did occur (not statistically detailed). | A short pre-op course of intranasal dexmedetomidine in patients with sleep disturbances significantly reduced POD incidence. Improved pre-op sleep (via DEX sedation) is posited as the mechanism, alongside anxiolysis. The DEX group had better sleep and much fewer cases of clinical insomnia pre-surgery, correlating with their lower delirium rate. Thus, treating preoperative sleep problems (e.g. with DEX) in at-risk cardiac surgery patients can decrease POD. |

^*^ Significant, ^≠^ not significant, CSA = central sleep apnea

** Incidence, AHI: apnea- hypopnea index, CAM: confusion assessment method, CABG: Coronary artery bypass grafting, CPB: cardiopulmonary bypass, DRS: Delirium Rating Scale, DOS: Delirium Observational Screening Scale, ESS: Epworth Sleepiness Scale, GSQS: Groningen Sleep Quality Score, HRV: heart rate variability, LSEQ: Leeds Sleep Evaluation Questionnaire, NEECHAM: Neelon and Champagne confusion scale, OSA: Obstructive sleep apnea, PSQI: Pittsburg sleep quality index, PROMIS: Patient-Reported Outcomes Measurement Information System, RCT: randomized controlled trial, RCSQ: Richards Campbell sleep questionnaire, REM: non-rapid eye movement, SA: sleep apnea, SE: sleep efficiency, SL: sleep latency, VSHSS: Verran and Snyder-Halpern Sleep Scale.

# PRISMA checklist

| **Section/topic** | **#** | **Checklist item** | **Location(s)**  **Reported** |
| --- | --- | --- | --- |
| **INFORMATION SOURCES AND METHODS** | | | |
| Database name | 1 | Name each individual database searched, stating the platform for each. | Search Strategy: page 4 |
| Multi-database searching | 2 | If databases were searched simultaneously on a single platform, state the name of the platform, listing all of the databases searched. | Table 1: page 5 |
| Study registries | 3 | List any study registries searched. | Search Strategy: page 4-5 |
| Online resources and  browsing | 4 | Describe any online or print source purposefully searched or browsed (e.g., tables of contents, print conference proceedings, web sites),  and how this was done. | Search Strategy: page 4-5 |
| Citation searching | 5 | Indicate whether cited references or citing references were examined, and describe any methods used for locating cited/citing references (e.g., browsing reference lists, using a citation index, setting up email alerts for references citing included studies). | Search Strategy: page 4-5 |
| Contacts | 6 | Indicate whether additional studies or data were sought by contacting authors, experts, manufacturers, or others. | Search Strategy: page 4-5 |
| Other methods | 7 | Describe any additional information sources or search methods used. | Search Strategy: page 4-5 |
| **SEARCH STRATEGIES** | | | |
| Full search strategies | 8 | Include the search strategies for each database and information source, copied and pasted exactly as run. | Table 1: page 5 |
| Limits and restrictions | 9 | Specify that no limits were used, or describe any limits or restrictions applied to a search (e.g., date or time period, language, study  design) and provide justification for their use. | Search Strategy: page 4 |
| Search filters | 10 | Indicate whether published search filters were used (as originally designed or modified), and if so, cite the filter(s) used. | Search Strategy: page 4 |
| Prior work | 11 | Indicate when search strategies from other literature reviews were adapted or reused for a substantive part or all of the search, citing  the previous review(s). | Search Strategy: page 4, 5 and Table 1 |
| Updates | 12 | Report the methods used to update the search(es) (e.g., rerunning searches, email alerts). | Search Strategy: page 4 |
| Dates of searches | 13 | For each search strategy, provide the date when the last search occurred. | Search Strategy: page 4 |
| **PEER REVIEW** | | | |
| Peer review | 14 | Describe any search peer review process. | - |
| **MANAGING RECORDS** | | | |
| Total Records | 15 | Document the total number of records identified from each database and other information sources. | Figure 1 |
| Deduplication | 16 | Describe the processes and any software used to deduplicate records from multiple database searches and other information sources. | Study selection: page 6 |

PRISMA-S: An Extension to the PRISMA Statement for Reporting Literature Searches in Systematic Reviews Rethlefsen ML, Kirtley S, Waffenschmidt S, Ayala AP, Moher D, Page MJ, Koffel JB, PRISMA-S Group.

Last updated February 27, 2020.

# References

1. Olds T, Blunden S, Petkov J, Forchino F. The relationships between sex, age, geography and time in bed in adolescents: a meta-analysis of data from 23 countries. Sleep Med Rev. 2010;14(6):371-8.

2. Jung DW, Hwang SH, Chung GS, Lee YJ, Jeong DU, Park KS. Estimation of sleep onset latency based on the blood pressure regulatory reflex mechanism. IEEE J Biomed Health Inform. 2013;17(3):534-44.

3. Reed DL, Sacco WP. Measuring Sleep Efficiency: What Should the Denominator Be? J Clin Sleep Med. 2016;12(2):263-6.

4. Shrivastava D, Jung S, Saadat M, Sirohi R, Crewson K. How to interpret the results of a sleep study. J Community Hosp Intern Med Perspect. 2014;4(5):24983.

5. Obstructive sleep Apnea. Illinois: American Academy of Sleep Medicine 2008.

6. Buysse DJ, Reynolds CF, 3rd, Monk TH, Berman SR, Kupfer DJ. The Pittsburgh Sleep Quality Index: a new instrument for psychiatric practice and research. Psychiatry Res. 1989;28(2):193-213.

7. Johns MW. A new method for measuring daytime sleepiness: the Epworth sleepiness scale. Sleep. 1991;14(6):540-5.

8. Bastien CH, Vallières A, Morin CM. Validation of the Insomnia Severity Index as an outcome measure for insomnia research. Sleep Med. 2001;2(4):297-307.

9. Locihová H, Axmann K, Žiaková K, Šerková D, Černochová S. Sleep quality assessment in intensive care: actigraphy vs. Richards-Campbell sleep questionnaire. Sleep Sci. 2020;13(4):235-41.

10. Richards KC, O'Sullivan, P. S., & Phillips, R. L. . Richards-Campbell Sleep Questionnaire (RCSQ) ) [Database record]. APA PsycTests. 2000.

11. Jafari B, Mohsenin V. Polysomnography. Clin Chest Med. 2010;31(2):287-97.

12. Rundo JV, Downey R, 3rd. Polysomnography. Handb Clin Neurol. 2019;160:381-92.

13. Iber C, American Academy of Sleep M. The AASM manual for the scoring of sleep and associated events : rules, terminology and technical specifications. Westchester, IL: American Academy of Sleep Medicine; 2007.

14. Sateia MJ. International classification of sleep disorders-third edition: highlights and modifications. Chest. 2014;146(5):1387-94.

15. Inouye SK, van Dyck CH, Alessi CA, Balkin S, Siegal AP, Horwitz RI. Clarifying confusion: the confusion assessment method. A new method for detection of delirium. Ann Intern Med. 1990;113(12):941-8.

16. Ely EW, Margolin R, Francis J, May L, Truman B, Dittus R, et al. Evaluation of delirium in critically ill patients: validation of the Confusion Assessment Method for the Intensive Care Unit (CAM-ICU). Crit Care Med. 2001;29(7):1370-9.

17. Marcantonio ER, Ngo LH, O'Connor M, Jones RN, Crane PK, Metzger ED, et al. 3D-CAM: derivation and validation of a 3-minute diagnostic interview for CAM-defined delirium: a cross-sectional diagnostic test study. Ann Intern Med. 2014;161(8):554-61.

18. Trzepacz PT, Baker RW, Greenhouse J. A symptom rating scale for delirium. Psychiatry Res. 1988;23(1):89-97.

19. Trzepacz PT, Mittal D, Torres R, Kanary K, Norton J, Jimerson N. Validation of the Delirium Rating Scale-revised-98: comparison with the delirium rating scale and the cognitive test for delirium. J Neuropsychiatry Clin Neurosci. 2001;13(2):229-42.

20. GUZE SB. Diagnostic and Statistical Manual of Mental Disorders, 4th ed. (DSM-IV). American Journal of Psychiatry. 1995;152(8):1228-.

21. Neelon VJ, Champagne MT, Carlson JR, Funk SG. The NEECHAM Confusion Scale: construction, validation, and clinical testing. Nurs Res. 1996;45(6):324-30.

22. Schuurmans MJ, Shortridge-Baggett LM, Duursma SA. The Delirium Observation Screening Scale: a screening instrument for delirium. Res Theory Nurs Pract. 2003;17(1):31-50.
